# Structural and dynamic properties of eutectic mixtures based on menthol and fatty acids derived from coconut oil: a MD simulation study

**DOI:** 10.1038/s41598-022-09185-x

**Published:** 2022-03-25

**Authors:** Samaneh Barani pour, Jaber Jahanbin Sardroodi, Alireza Rastkar Ebrahimzadeh, Mohammad Sadegh Avestan

**Affiliations:** 1 Molecular Simulation Lab, Azarbaijan Shahid Madani University, Tabriz, Iran; 2 Department of Chemistry, Azarbaijan Shahid Madani University, Tabriz, Iran; 3 Department of Physics, Azarbaijan Shahid Madani University, Tabriz, Iran; 4 Molecular Science and Engineering Research Group (MSERG), Azarbaijan Shahid Madani University, Tabriz, Iran; 5 Department of Chemistry, University of Cincinnati, Cincinnati, OH 45221 USA

**Keywords:** Chemistry, Engineering

## Abstract

The structural and dynamical properties of the binary mixture of Menthol (MEN) and Fatty acids (FAs) were investigated using molecular dynamics simulations. To this end, the relationship between the structural and dynamical properties of the eutectic mixtures of MEN and FAs with different molar percentages of FAs are studied. Structural properties of the eutectic mixtures were characterized by calculating the combined distribution functions (CDFs), radial distribution functions (RDFs), angular distribution functions (ADFs), hydrogen bonding networks, and spatial distribution functions (SDF). Additionally, our Results indicated robust interactions between menthol and Caprylic acid molecules Finally, the transport properties of the mixtures were investigated using the mean square displacement (MSD) of the centers of mass of the species, self-diffusion coefficients and vector reorientation dynamics (VRD) of bonds. Overall, our simulation results indicated that intermolecular interactions have a significant effect on the dynamic properties of species.

## Introduction

Menthol [1-meMENl-4-(1-meMENleMENl) cyclohexan-3-ol] is a cyclic terpene alcohol that may be could be extracted from the oil of plants of the Mentha genus^1^. This natural product has been used for medicinal purposes, cosmetics, as a flavor and, as an intermediary in the production of widely used industrial solvents, such as deep eutectic solvents^2^.

The deep eutectic solvents (DESs) that were introduced by Abbot et al. for a mixture of choline chloride and urea^4^, are formed using strong interactions between the hydrogen bond donor (HBA) and hydrogen bond acceptor (HBD)^3^. Eutectic mixtures have melting points much lower than their individual components. The volatile organic solvents, such as including methanol, ethanol, and chloroform^5^, due to their extensive usage in the industry, give rise to various diseases. Therefore, to ensure better human health, ionic liquids (ILs) have attained great attention as an alternative to these toxic solvents including methanol, ethanol, and chloroform^5^. ILs are renowned for being non-flammable, water-compatible, and biocompatible. It has been found that DESs and ILs have similar physical characteristics such as viscosity, conductivity, and surface tension^6^. DESs are known as green solvents, which are mostly preferred in the industry due to their biodegradability, lower toxicity, and reasonable price^6^. The diverse applications of eutectic solvents in chemical processes and the removal of pollutants from the environment represent the DESs as a great candidate for various applications. So far, the most widely proposed eutectic solvents in the open literature are the DESs prepared from hydrophilic compounds, such as sugars, organic acids, sugar alcohols, and amino acids with choline chloride. The hydrophilic eutectic solvents are freely miscible with water; thus, they are unstable inside water^7^.

Since 2015, Hydrophobic DES was receiving a great amount of has been studied extensively due to the stable hydrophobic phase in contact with water and inexpensive and natural components that follows the principles of green chemistry^5^. Previous studies show that the hydrophobic DESs based on quaternary ammonium halides salt are able to extract of hydrophobic phytochemicals, such as artemisinin and polyprenyl acetates, from plant materials^8, 9^. One of the major challenges in the extraction of the extracted materials from plants is extraction with non-toxic solvent. Marrucho et al. have reported several Hydrophobic deep eutectic solvents composed of natural, nontoxic compounds, i.e., terpenes and fatty acids^10^. Menthol-based hydrophobic DESs have lower viscosity and higher volatility than hydrophobic quaternary-ammonium based DESs^5^. Furthermore, it is easy to recover target compounds from menthol based DESs. Eutectic mixtures of menthol and saturated fatty acids can be derived from coconut oil and palm kernel oil^11^. Some Menthol-based Eutectic Mixtures have very interesting applications in the extraction of harmine and biomolecules such as caffeine, tryptophan, isophthalic acid, and vanillin from the aqueous phase^10^. In addition, the DES composed of the menthol and acetic acid has been used to extract phytocannabinoids from raw cannabis plant material^12^.

Because eutectic solvents have been widely used in the isolation of pollutants from aqueous solutions, it is critical to investigate their thermophysical and structural properties at different temperatures. Because DES properties are controlled by nanoscopic factors, understanding the nanoscopic factors enables tuning of molecular properties to achieve the desired macroscopic properties^13^. Molecular dynamics simulation is a valuable tool, which would provide insight into the eutectic mixtures at the molecular level in modern chemistry and chemical engineering^14^. Therefore, can a suitable appropriate in connecting nanoscopic features to macroscopic physicochemical properties. However, the parameters (e.g., in the CHARMM force field) that are mandatory to design of FAs molecules and predict their physicochemical properties are not reported. As a result, in the present study the force field parameters and partial atomic charges were computed using quantum calculations. QM optimizations of force field parameters were considered for MD simulations. We were particularly interested in the effect of the percentage composition of species and temperature on the dynamic and structural properties of the eutectic solvents based on menthol and fatty acid (see Supplementary Table S5). The intermolecular interactions that can be used to predict macroscopic physicochemical properties have been identified. The structural and dynamic properties of eutectic solvents can provide a valuable insight inro the intermolecular interactions that can cause a significant drop in the melting point of the binary mixtures.

Despite experimental studies on the physical properties of DESs based menthol an extended molecular dynamics simulation studies on the effect of the percentage composition of species and temperature on the dynamic and structural properties of the binary mixtures based on menthol and fatty acid, which can provide us with more information in the microscopic level, has been missing to date. To this end, in this study binary mixtures of Caprylic Acid: Menthol, Decanoic acid: Menthol, and Lauric Acid: Menthol were prepared and MD simulations were performed for exploring the interplay between dynamical properties and local structures in binary mixtures. The obtained densities for the binary mixtures of FAs and MEN in our study are comparable to the experimental results^8^. We hope that the obtained parameters in the present study will be useful for the thermos-physical and dynamic properties that have not been reported so far. From the CDFs composed of RDF and ADF, the convenient sites for the hydrogen bond interactions between MEN and FAs were determined. The results showed that the strong hydrogen bond interactions are formed between the oxygen atoms of FAs and the hydrogen atoms of MEN at the distance range of 2 to 3 Å and the angle range of 130° to 180°. The stability of the H-bond between the two species can lead to decreasing of self-diffusion coefficients of the species in the binary mixtures of MCA, MDA, and MLA with %FAs = 44, 35, and 25, respectively and they are in agreement with the obtained SDFs.

## Computational methods

Three acids such as Caprylic acid (CAP), Decanoic acid (DEC), and Lauric acid (LUA) were selected in the saturated fatty acids for MD simulation. PACKMOL package was used for the preparation of the binary mixtures of MEN and FAs with the different molar percentages of FAs^11^. The information about the simulated boxes and their different combinations (HBD and HBA) with abbreviations are listed in Table 1. The initial simulation box included with the mole percent of FAs at 20.0% and 80.0% of MEN was provided using random distribution. In the next step, the number of FAS molecules was increased for the preparation of the mixtures with the mole percent of FAs at 70.0%. The NAMD 2.13 software package was used to perform all the MD simulations^15^. First, all the initial configurations were minimized to remove the bad contacts and then the binary mixtures were heated to 323 K to prepare a uniform liquid phase. In the next steps, an NVT ensemble (constant number of particles, volume, and temperature) was performed for 5 ns at 323 K which is followed by the equilibration of the binary mixture for 50 ns in the NPT ensemble (constant number of particles, pressure, and temperature) with a time-step of 1 fs. After equilibrating the systems, its liquid phase was analyzed at 353 K. The binary mixtures with the most intermolecular interaction between HBD and HBA were selected, and their temperature was increased slowly from 0 to 353 K and then decreased back to 323 K and 300 K with a temperature gradient of 1 K/FS. The Nose–Hoover thermostat^16^ and the Parrinello–Rahman pressure coupling^17^ were used to maintain a constant temperature and pressure, respectively, during the simulation. Periodic boundary conditions were imposed or the simulated systems. Force field parameters and partial atomic charges were calculated by fitting the RESP computed at the MP2/6-31G* level^18^. The applied total potential energy has the following different terms:

**Table 1:** Names of the simulated eutectic solvents and their different combinations (HBD and HBA) with abbreviations, and the MEN: FAs ratios in the binary mixtures.

Name	HBD	HBA	%FAs	n_MEN_:n_FAs_
MCA	Caprylic acid (CAP)	Menthol (MEN)	20, 44, 70	800:200, 560:440, 300:700
MDA	Decanoic acid (DEC)	Menthol	20, 35, 70	800:200, 650:350, 300:700
MLA	Lauric acid (LUA)	Menthol	20, 25,70	800:200, 750:250, 300:700

$$E_{{bonded}} = \sum\limits_{{bonds}} {k_{b} } \left( {b - b_{0} } \right)^{2} + \sum\limits_{{angles}} {k_{\theta } } \left( {\theta - \theta _{0} } \right)^{2} + \sum\limits_{{\begin{array}{*{20}c} {improper} \\ {dihedrals} \\ \end{array} }} {k_{\varphi } \left( {\varphi - \varphi _{0} } \right)^{2} } + \sum\limits_{{dihedrals}} {\sum\limits_{{n = 1}}^{6} {k_{{\emptyset ,n}} } } (1 + {\text{cos}}\left( {n\emptyset - \delta _{n} )} \right)$$

1 $$E_{{nonbonded}} = \sum\limits_{{\begin{array}{*{20}c} {nonbonded} \\ {pairi,j} \\ \end{array} }} {\frac{{q_{i} q_{j} }}{{4\pi D\left| {\left| {r_{i} - r_{j} } \right|} \right|}}} + \sum\limits_{{\begin{array}{*{20}c} {nonbonded} \\ {pairi,j} \\ \end{array} }} {\epsilon _{{ij}} \left[ {\left( {\frac{{R_{{min,ij}} }}{{\left| {\left| {r_{i} - r_{j} } \right|} \right|}}} \right)^{{12}} - 2\left( {\frac{{R_{{min,ij}} }}{{\left| {\left| {r_{i} - r_{j} } \right|} \right|}}} \right)^{6} } \right]} .$$

This force field includes bonded parts such as bonds, angles, and torsional dihedral interactions and the non-bonded interactions consisting of van der Waals (vdW) and electrostatic (Eel) terms^18^. The particles mesh Ewald (PME) method and the Lennard–Jones (LJ) 6–12 potential with a cut-off distance of 12 Å were applied for long-range and short-range interactions, respectively^18^.

## Results and discussion

### Radial distribution functions (RDFs)

**Figure 1:**
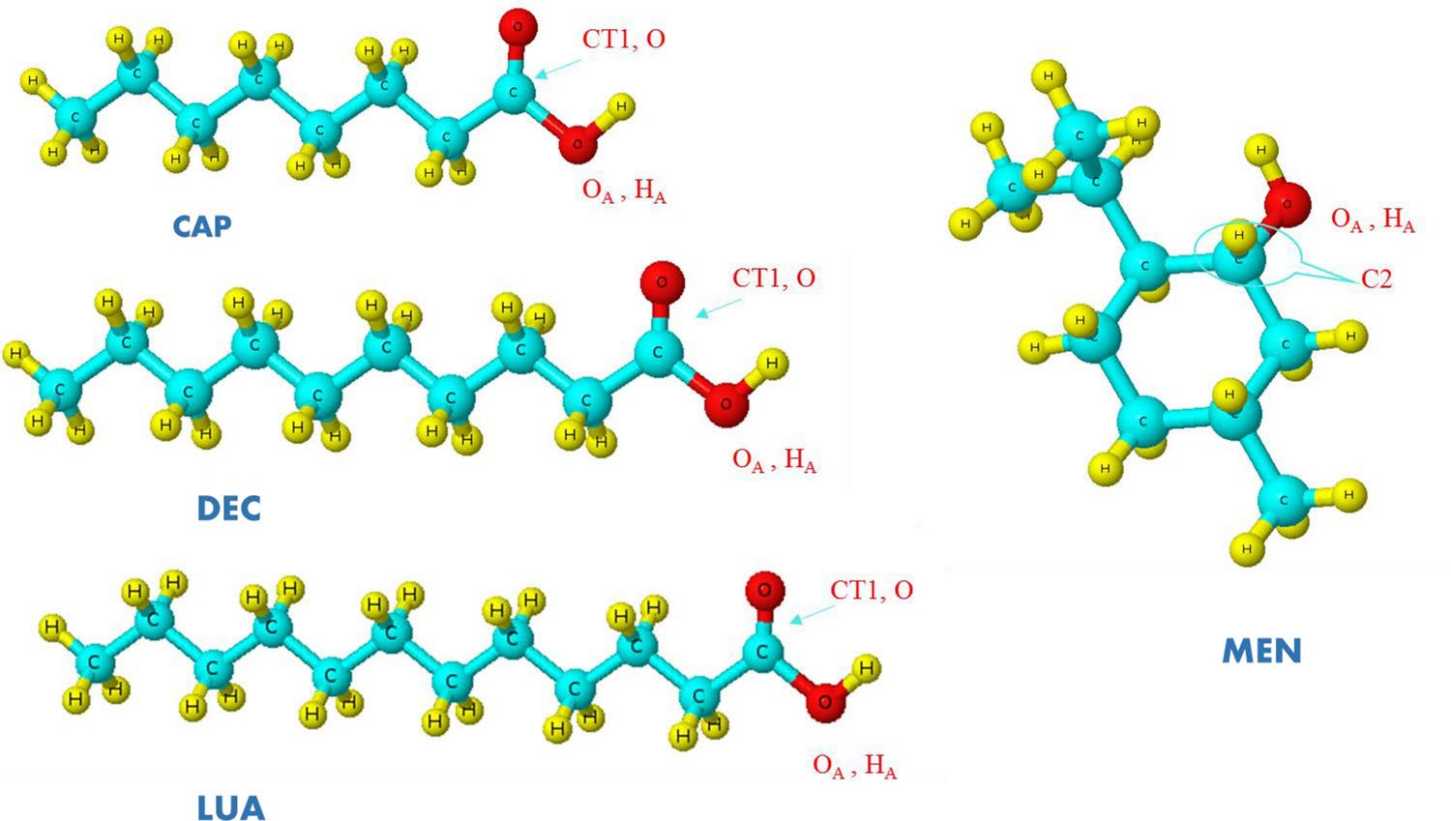
Schematic of Menthol (MEN) and Fatty acids (FAs) with the main labels.

**Figure 2:**
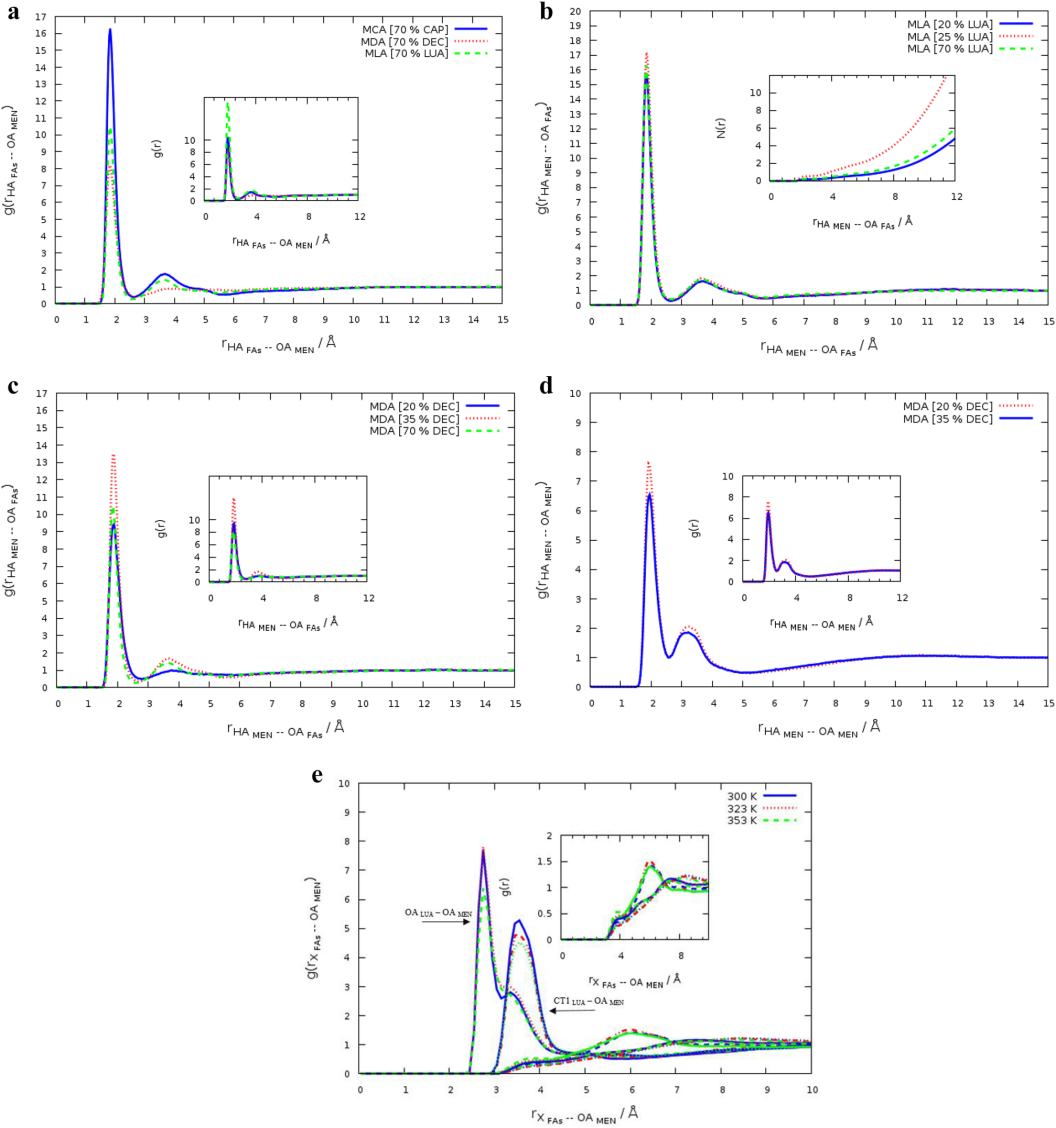
Atom–atom RDFs for Fas–MEN, (**a**) RDFs between HA atom FAs and the OA atom on the menthol molecules in the binary mixtures with %FAs = 70 at 323 K, (**b**) RDFs between HA atom on the menthol molecules and the OA atom of LUA in the binary mixtures with % LUA = 20, 25, and 70 at 323 K, (**c**) RDFs between HA atom on the menthol molecules and the OA atom of DEC in the binary mixtures with % DEC = 20, 35, and 70 at 323 K, (**d**) RDFs between menthol molecules, g(r)_MEN–MEN_, for the binary mixtures of MEN and DEC with % DEC = 20 and 35 at 323 K, (**e**) Site–site RDFs between atoms of the LUA molecules and the OA on the menthol molecule in the binary mixtures at 323 K.

The radial distribution function (RDF) can represent how the average species density varies as a function of distance from a given species within the system^18^. This quantity was computed to determine the average distribution of species around any given species the^18^. g(r) is given by

2 $$g\left( r \right) = \frac{1}{{\rho N}}\left\langle {\sum\limits_{{ij}} {\delta \left( {r - r_{{ij}} } \right)} } \right\rangle ,$$

where N indicates the number of species in the system, $\rho$ is the density of species^18^. The angle brackets are an average over all the species. This information could be used to characterize various interactions between species in the binary mixtures of MEN and FAs. To better understand the structural properties of the binary mixtures, RDFs of the H atoms of HBD around the O atoms of the HBA was obtained. Figure 1 depicts labels of the atoms in the hydrogen bond donor and acceptor. The Fas–MEN RDFs have a main peak and a small plateau in the vicinity of the first maximum peak, while all RDFs between menthol molecules have a similar shape with two maxima. The sharp peaks of HA_Fas_–OA_MEN_ RDFs are clear indications of interaction between the hydroxyl hydrogen of FAs and the O atom of MEN at 2 Å distance (see Fig. 2a). The pair interaction of two species has more intensity in the binary mixtures of MEN and with % CAP = 44, % DEC = 35, and % LUA = 25 (see Fig. 2b,c). The RDFs between menthol molecules were investigated in the binary mixtures with different percentages of FAs molecules. RDFs between menthol molecules in the binary mixtures with % DEC = 20 and 35 are shown in Fig. 2d. The shoulder and the main peak are located at about 3 and 2 Å in the binary mixtures of DEC and MEN. The height of the first peak of the MEN–MEN RDF gradually was decreased with increasing the mole percent of FAs. These results indicate that the probability distribution of menthol molecules around FAs molecules increases in the binary mixtures.

**Figure 3:**
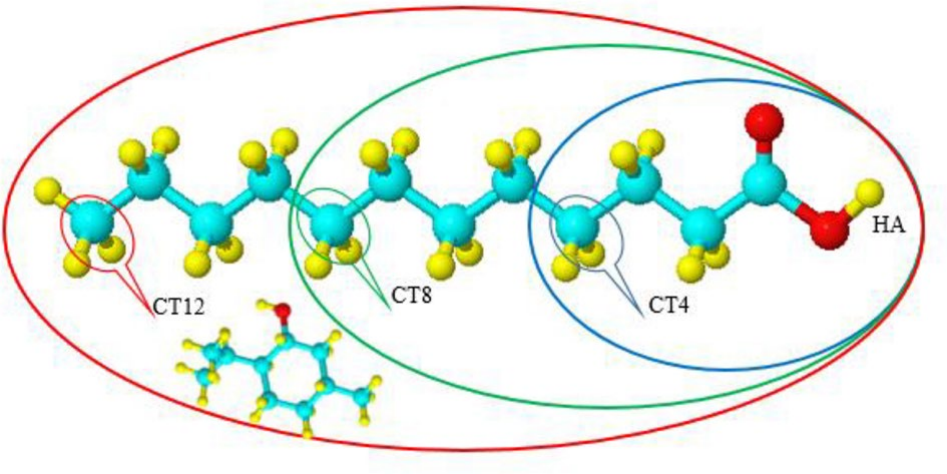
The regions of the LUA molecule in the binary mixture: CT4 (blue ellipse), CT8 (green ellipse), and CT12 (red ellipse).

To investigate the effect of temperature on the site–site RDFs between the CTn–CTn tails (n = 2, 4, 8, and 12 corresponding to the number of carbon atoms of the alkyl chain of FAs) and the carboxylic head of FAs with the OA atom of MEN and were analyzed at different temperatures (see Fig. 3). The site–site RDFs of the different atoms of FAs with the OA atom of MEN is shown in Fig. 2e. The position of the maximum peaks of RDFs between the C atoms of the alkyl chain and the OA atom of MEN are located at farther distances in comparison to the RDFs of the carboxylic head of FAs and the OA atom of MEN. The first peak of HFAs–OAMEN RDFs is much sharper than the first peaks of RDFs between the C atoms of the alkyl chain and the OA atom of MEN. Comparing the peaks at different temperatures, the results show that there is a strong interaction between the HA atom of FAs and the OA atom of MEN at 300 K. The distribution of the C atoms of FAs in the nonpolar region around the OA atom of MEN is obviously different at various temperatures. For distances more than 6 Å, the height of the first peak of the RDFs in various temperatures are almost similar. RDFs between the CTn–CTn tails and the OA atom of MEN indicate that the CT1–CT1 aggregation degree around the OA atom of MEN is reduced by increasing of temperature. Due to reorientation dynamics of the bond vectors, the coordination number of the RDFs peaks at 353 K is less commonly seen than the CN of the RDFs at 300 K.

#### Coordination number

**Figure 4:**
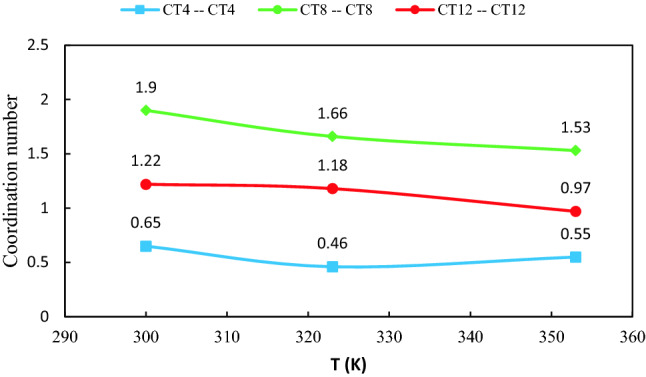
Coordination numbers of tails CTn–CTn around the OA atom of MEN in the binary mixtures of LUA and MEN.

The coordination number is defined as the number of molecules under the first peek of the RDF curve between a given molecule and central molecule of the mixture^19^. Generally, the CN can be gained by integrating the surface region surrounded at the first solvation shell (rfs). The CNs are calculated by Eq. (3)***.***

3 $$N = 4\pi \rho \int\limits_{0}^{{r_{{sell}} }} {r^{2} g\left( r \right)dr} ,$$

where $\rho$ and ${r}_{sell}$ represent density and the first minimum in the RDFs, respectively^20^. Coordination numbers of the molecules around each other in the binary mixtures are listed in Supplementary Table S1. Our results indicated that the first solvation shell of the MEN–MEN RDF molecules in the binary mixtures of MCA, MDA, and MLA with %FAs = 44, 35, and 25, respectively, accommodated a smaller number of the menthol molecules compared to the other range of %FAs. Decreasing the aggregation degree of the MEN molecules around each other can be attributed to the strong interactions between the two species in the binary mixtures. Most likely, the FAs have been acted as hydrogen-bond acceptors and hydrogen-bond donors in the binary mixtures. Dual roles of both HBA and HBD of acids in the binary mixtures with %FAs = 70 is noticeably higher than other ratios. The reduction of the distribution of acid molecules around each other in mixtures with %FAs = 44, 35, and 25 can be thought of as some evidence of this result. To further study temperature dependence, the site–site RDFs between the LUA and the OA atom of MEN were calculated at different temperatures. The CNs of the carbon atom of the alkyl chain (CTn–CTn) are shown in Fig. 4. In general, the CNs are reduced with increasing temperature. The coordination number of CT12 around menthol was notably greater than the CT4 in the binary mixtures of MLA. This aggregation is the result of the van der Waals force and it can be also due to lack of many hydrogen bonds. The competition between electrostatic and van der Waals interactions leads to the aggregation via the carbon atom (CTn) of the tail.

### Hydrogen bond analysis

**Figure 5:**
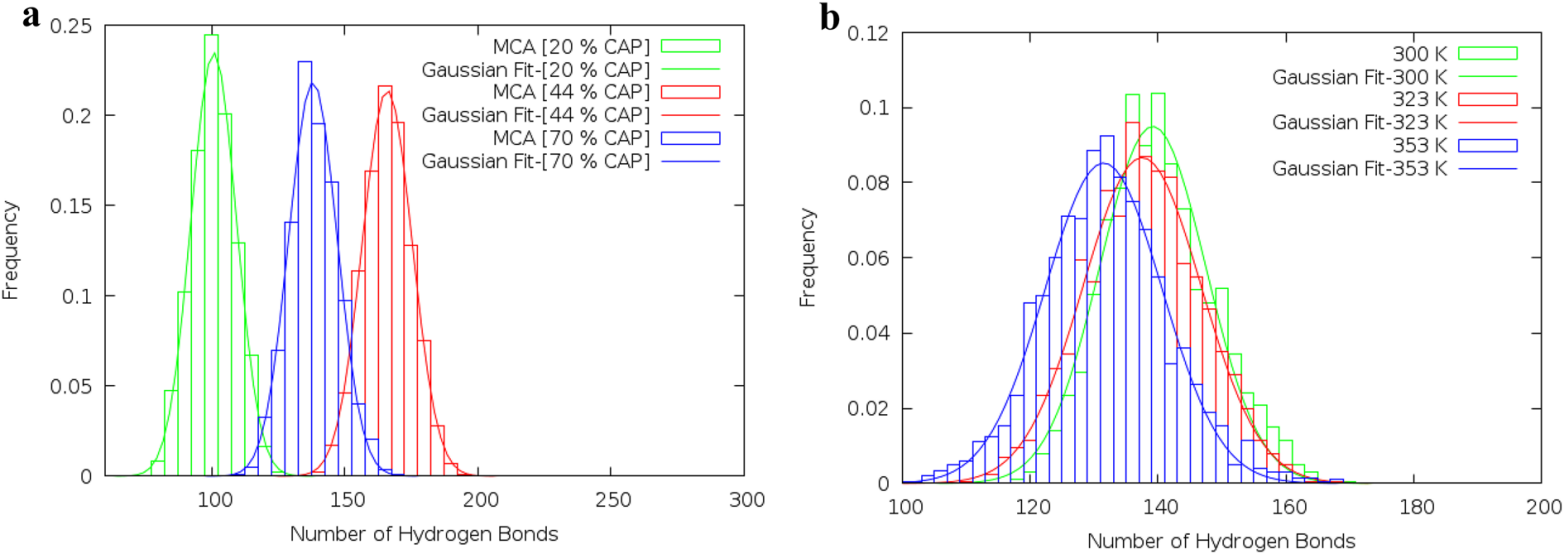
(**a**) The distribution of the hydrogen bond between menthol and CAP molecules in the binary mixtures with %CAP = 20, 44 and 70 at 323 K, (**b**) The distribution of the hydrogen bond between menthol molecules in the binary mixture with %LUA = 44 at different temperatures.

**Figure 6:**
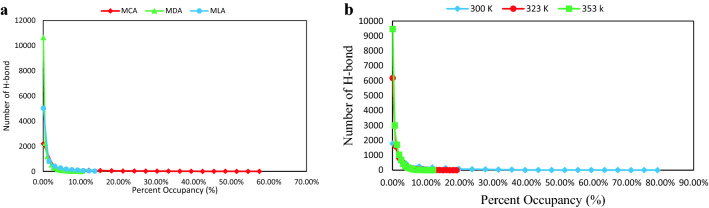
(**a**) Hydrogen bond percent occupancies between the H atoms of MEN and the O atom of FAs in the binary mixture with a molar percentage of 70% at 323 K, (**b**) Hydrogen bond percent occupancies between the H atoms of MEN and the OA atom of LUA in the binary mixture with a molar percentage of 25% at different temperatures.

In the eutectic mixtures, strong D–H⋯A hydrogen bonding leads to a reduction in the melting point of the binary mixtures. Hydrogen bond criteria are defined based on distance and angle. Statistically, the probability of the observed exact value of the distance and angle is zero. According to the suggestions of Wernet et al., the set of two separate criteria (one distance and one angle) from the specific region of CDF plots were selected to determine the presence of a (strong) H-bond^21^. The combined distribution functions (CDFs) involving the radial distribution functions (RDFs) and the Angular Distribution Function (ADF) can be computed with TRAVIS. The selected distances and angles are given below: 2 Å (150°) for HA_MEN_–O_CAP_, 2 Å (135°) for HA_CAP_–O_CAP_ and 2.25 Å (150°) for HA_MEN_–OA_MEN_ pairs (see Fig. 8a,d,e). Menthol molecule with the atom labeling scheme is given in Supplementary Fig. S1. The number of H-bonds between different species (HBD and HBA) was measured over time. The average number of hydrogen bonds is obtained via fitting of Eq. (4)

4 $$F\left(X\right)={\frac{a}{\sigma \sqrt{2\pi }}exp}^{\frac{{-(X-\overline{X })}^{2}}{2{\sigma }^{2}}},$$

where σ, and $\overline{X }$ are the standard deviation and the average number of hydrogen bonds, respectively^22^. The average number of hydrogen bonds between species is an important parameter for the formation of eutectic solvent that changes under the influence of the relative percentage of species and temperature. The hydrogen bonds in the binary mixtures of MCA, MDA, and MLA with %FAs = 44, 35, and 25 are more than hydrogen bonds in the mixtures with other ratios (see Supplementary Table S2). The H-bond number between FAs and MEN was reduced in the binary mixtures with a higher percentage of FAs. Most likely, Decreasing the average number of hydrogen bonds is due to the distribution of the number of hydrogen bonds between the carboxyl groups of FAs (see Fig. 5a). The hydrogen-bonding network is directly affected by the temperature of the mixture. Investigation of the hydrogen bonding network in the binary mixture at the temperature range of 300 to 353 also showed that the H-bond number was slightly decreased with raising of the temperature (see Fig. 5b). The total time that a hydrogen-bond network has established between HBD and HBA is described using the percent occupancy^23^. This analysis has depicted the stability and persistence of hydrogen bonds between the possible HBD and HBA pairs^24^. The stability of the H-bonds interactions between different atoms of the MEN molecules and the O atom of FAs molecules was investigated in the binary mixture. The hydrogen-bonded network between the HA atom of MEN and the O atom of FAs is more stable than the other atoms of MEN. Furthermore, interactions between the HA atoms of FAs and the OA atom MEN have the highest percent occupancy of hydrogen bonding. The stability of the hydrogen bonding network between the different species is provided in Fig. 6a,b. Figure 6a displays the Hydrogen bond occupancy percentage of the HA atoms of MEN and the O atom FAs in the binary mixture with a molar percentage of 70% at 323 K. The percent occupancy of the hydrogen bonding between HBD and HBA was the highest in the binary mixture of MCA. The stability of the H-bond between the two species was decreased by increasing the carbon chain length of FAs from C8 to C12. The occupancy of the hydrogen bonding between three kinds of HBA and MEN has the highest content in the binary mixtures of MCA, MDA, and MLA with %FAs = 44, 35, and 25, respectively, the number of H-bonds between different species (HBD and HBA) were measured over time. We surmise that, the strong interaction between FAs and MEN at 300 K is due to hydrogen bonding with the highest percentage of occupancies. The stability of the hydrogen bonding network confirmed density reduction trend of the mixture at distinct temperatures (see Supplementary Table S4).

### Spatial distribution functions (SDFs)

**Figure 7:**
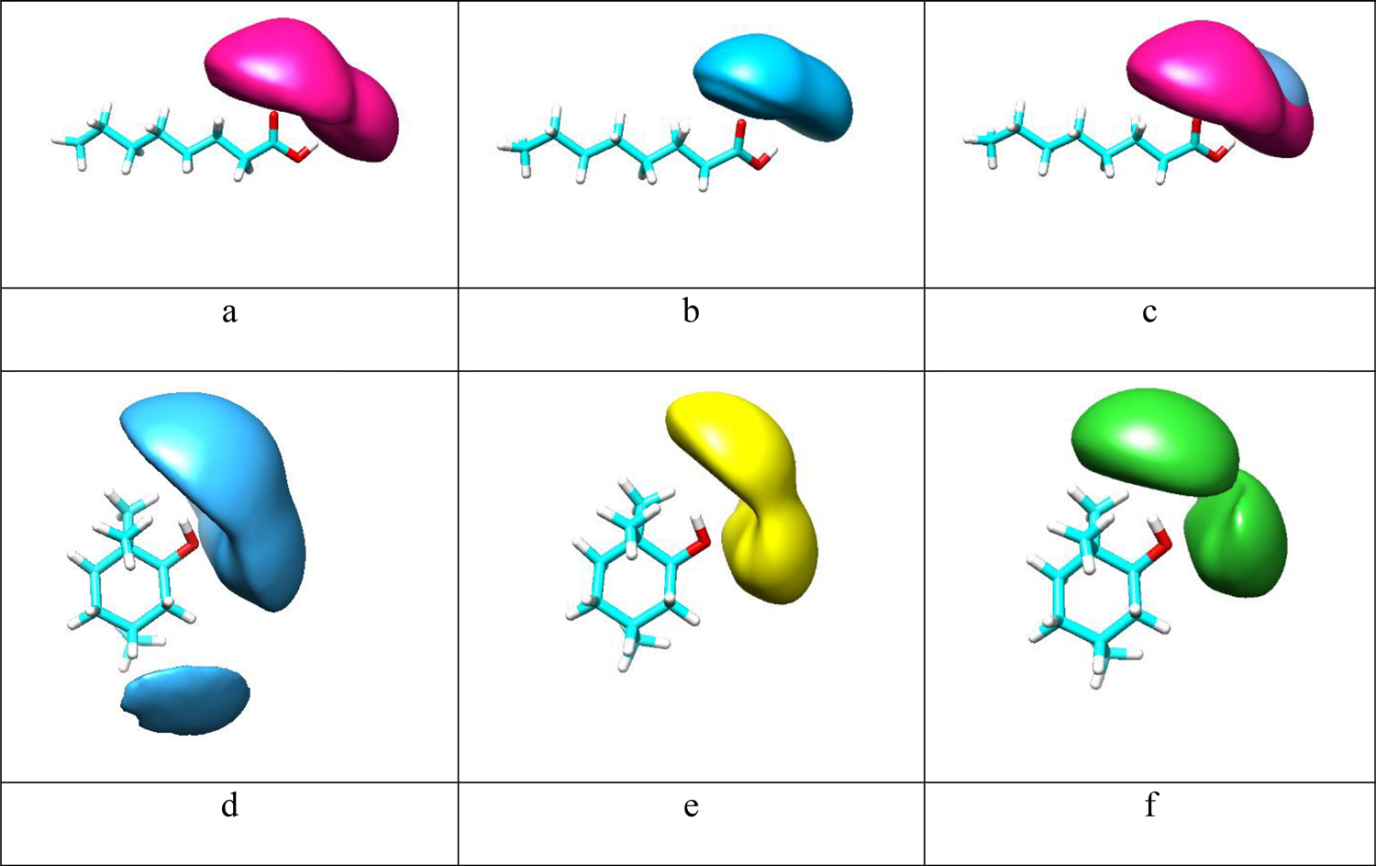
Spatial distribution functions (SDFs) of the binary mixtures with the mole percent of FAs at 70% at 323 K. Green isosurfaces correspond to LUA, yellow isosurfaces correspond to DEC, blue isosurfaces are CAP molecules, pink isosurfaces are MEN molecules. The reference molecules are Caprylic acid (**a**–**c**), or MEN molecule (**d**–**f**).

For further visualization of the local structure of the eutectic mixture, spatial distribution functions (SDFs) were analyzed^25^. SDFs can demonstrate the desirable sites for intermolecular hydrogen-bond interactions. Three-dimensional spatial distribution functions of different species around a reference molecule were plotted in Fig. 7a–f. The distribution of menthol molecules around the Carboxylic acid group of FAs is seen vividly from the isosurfaces Fig. 7a–c. The density distribution function (Dens) of the HA atom of MEN around the OA atom of FAs was investigated in the binary mixtures and the obtained values are represented in Table 3. The values of the Dens were determined using the first minimum peak of the corresponding particle density curve. The values of the Dens between the HA atom of MEN and the OA atom of CAP are 0.25, 0.19, and 0.15 in the binary mixtures with %FAs = 44, 35, and 25, respectively. The maximum number of hydrogen bonds (H-bond) of the two species around each other of approximately 201.21 was observed in the binary mixtures with %CAP = 44. Therefore, the results were confirmed using Hydrogen bonds analysis. These observations are also in agreement with the results of the combined distribution function. Furthermore, the probabilities of the FAs molecules around the hydroxyl group of MEN molecules were decreased by increasing the length of the alkyl chain of FAs (see Fig. 7d–f). SDFs of acid molecules around each other showed that these molecules are mainly distributed around each other in H–O and O=C sites of the carboxyl group. We remark that the local density of the HA atom of FAs appears more distant than the density of MEN molecules around CAP molecules. In the mole percent of FAs at 70%, the reduction of the MEN molecules distribution around the FAs molecules in the binary mixtures is more likely associated with the accumulation of acid molecules around each other. The maximum distribution between the two species of MEN and FAs is not generally the same at different temperatures. Increasing temperatures cause a shift in the aggregation behavior of molecules around each other. At high temperatures, it appears that the stability of intermolecular hydrogen bonds decreases. The stability of intermolecular hydrogen bonds may affect the local solvation structure in the binary mixtures. It should be noted that the isovalues were selected to reflect the completion of the first solvation shell^26^.

### Tracing [MEN] [FAs] H-bonds using combined distribution function (CDF)

**Figure 8:**
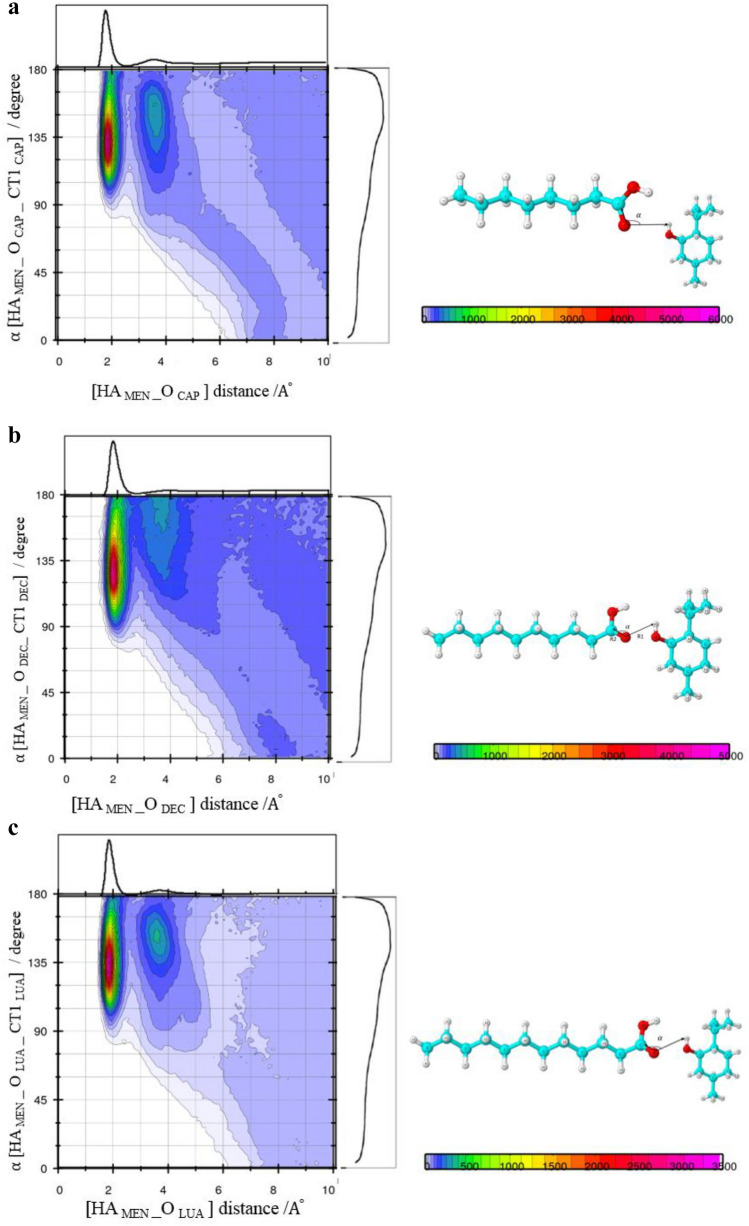

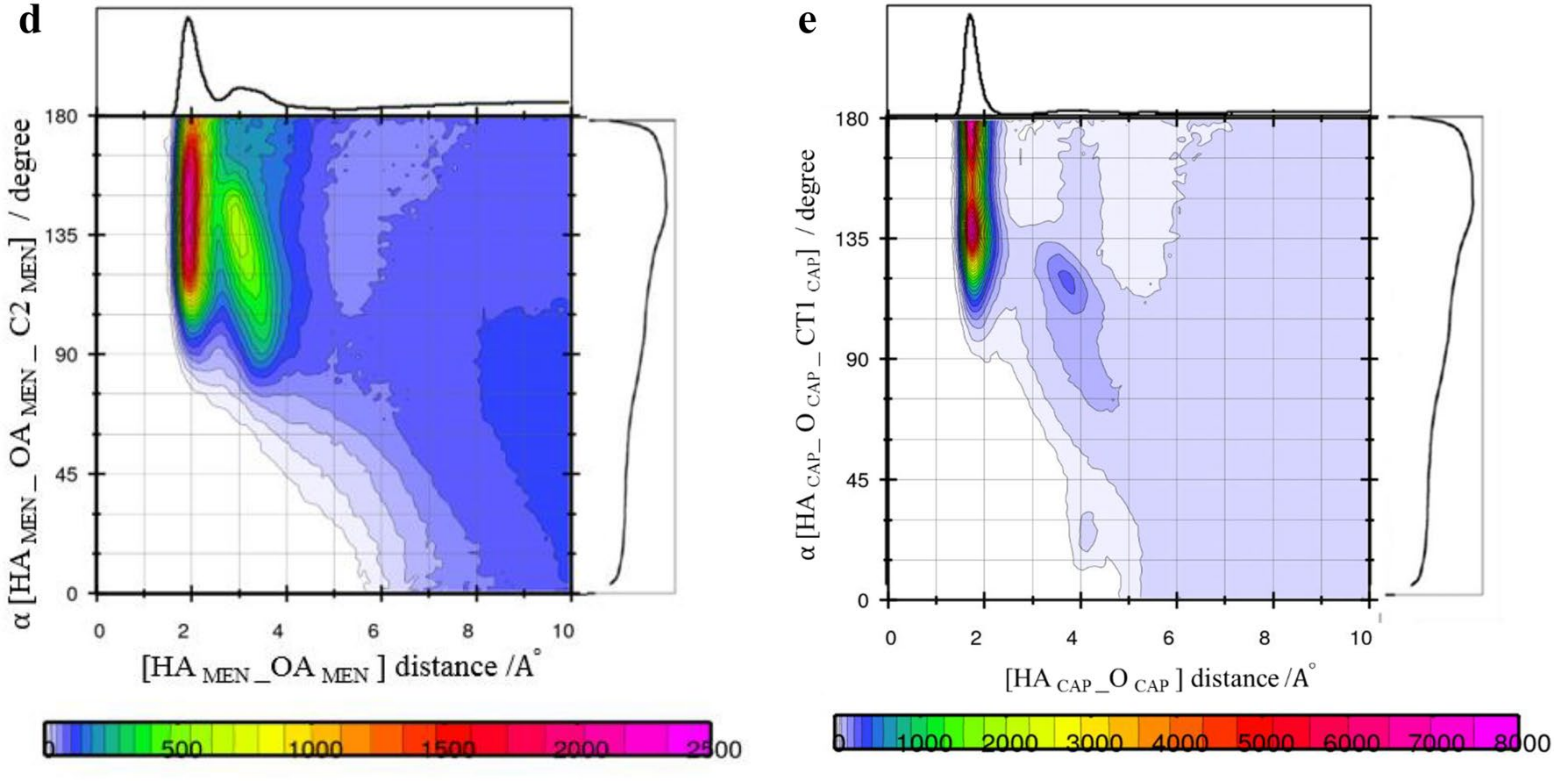
Combined radial/angular distribution functions for, (**a**) the HA_MEN__O_CAP_ distance and HA_MEN__O_CAP__CT1_CAP_ angle, in the binary mixtures with %CAP = 70 at 323 K, (**b**) the HA_MEN__O_DEC_ distance and HA_MEN__O_DEC__CT1_DEC_ angle, in the binary mixtures with % DEC = 70 at 323 K, (**c**) the HA_MEN__O_LUA_ distance and HA_MEN__O_LUA__CT1_LUA_ angle, in the binary mixtures with %LUA = 70 at 323 K, (**d**) the HA_MEN__OA_MEN_ distance and HA_MEN__OA_MEN__C2_MEN_ angle, in the binary mixtures with % CAP = 44 at 323 K, (**e**) the HA_CAP__O_CAP_ distance and HA_CAP__O_CAP__CT1_CAP_ angle, in the binary mixtures with % CAP = 44 at 323 K.

The distance criteria of the H-bond were determined from the first minimum of the peaks of the corresponding RDFs in the binary mixtures. However, the distance criteria may not be adequate to capture the presence of a strong H-bond in the binary mixtures^21^. Therefore, the combined radial/angular distribution functions were obtained for all possible pairs. The composed CDFs of the RDF between the hydroxyl hydrogen (HA) site of MEN and the O atom of the FAs as the X-axis, the Angular Distribution Function (ADF) between two vectors (R1 and R2) as the Y-axis are shown in Fig. 8a. H-bonds (weak or strong) were found at the angles range between 130° and 150° and distances of less than 2 Å. Figure 8a shows that there are more menthols around CAP than the other acids in the binary mixtures (see Fig. 8b,c). CDFs analysis also displays strong H-bond interaction, i.e. –OH…O=C in the binary mixtures of MCA, MDA, and MLA with %FAs = 44, 35, and 25, respectively. The highest angular/distance probability region at around 130°–160°/2 Å fulfills the geometric conditions of H-bond formation for the (HA–OA) menthol–menthol structural correlation.

**Figure 9:**
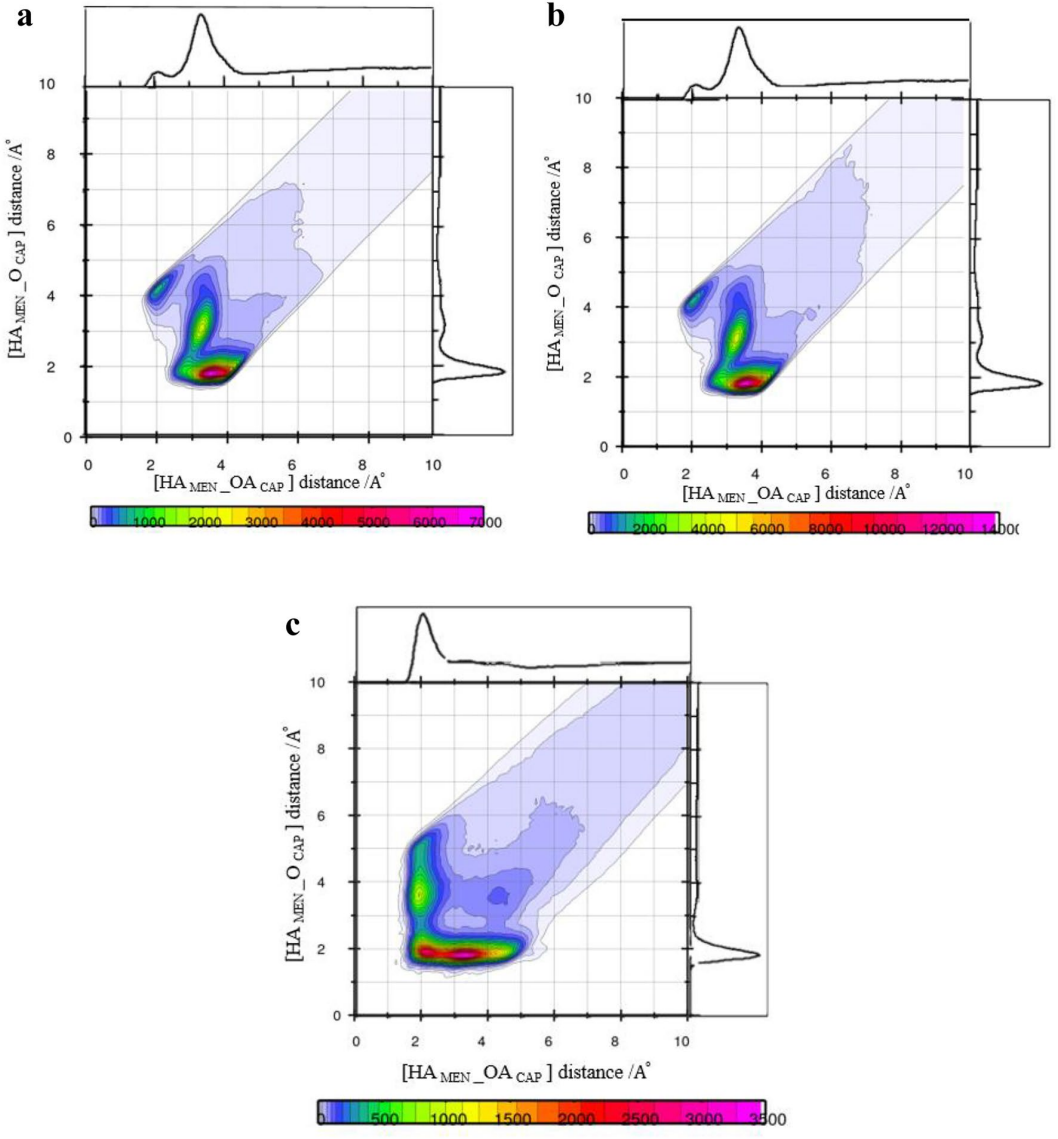
(**a**) Combined radial/radial distribution functions for (**a**) the HA_MEN__O_CAP_ distance and HA_MEN__OA_MEN_ distance, in the binary mixtures with % CAP = 20 at 323 K, (**b**) for the HA_CAP__O_CAP_ distance and HA_CAP__OA_MEN_ distance, in the binary mixtures with % CAP = 44 at 323 K, (**c**) the HA_CAP__O_CAP_ distance and HA_CAP__OA_MEN_ distance, in the binary mixtures with % CAP = 70 at 323 K.

In the binary mixtures with the same mole percent of FAs, the presence of CAP was greatly inhibited strong interactions between MEN molecules compared to other FAs. Respective distance/distance CDFs including three neighbor molecules (MEN…FAs…MEN) were computed to gain a better understanding of the bridging role of species in binary mixtures. The possibility of connecting two MEN molecules as the HBD by one [FAs] bridge was investigated in the binary mixtures with the different mole percentage of FAs. The respective CDF includes two axes in which the x-axis illustrates the distance between the H atom of the MEN, HA, and carboxyl oxygen of the neighbor FAs, OA, and the y-axis illustrates the distance of fatty acid-menthol neighbors (O–HA). The intense probability was observed at distances of 2 Å and 3 Å. The results showed that the carboxyl group of [FAs] can bind two MEN molecules in the binary mixtures (see Fig. 9a–c). The occurrence probability of the [MEN]···[FAs]···[MEN] structure in the binary mixtures with a molar percentage of 70% was attenuated due to the H-bonding formation between FAs molecules.

### Non-bonded interaction energy

The non-bonded interaction energy is an important analysis for understanding intermolecular interactions in the binary mixtures. The non-bonded energy includes two terms, short-range van der Waals (vdW) interactions and long-range electrostatic interactions, which are calculated using Lennard–Jones (12–6) function and Coulombic equation, respectively^27^. The vdW and Coul interactions are described by the following equations:

5 $$E_{{nonbonded}} = E_{{Coulombic}} + E_{{vdW}} = \frac{{e^{2} }}{{4\pi \varepsilon _{0} }}\sum\limits_{{i \ne j}} {\frac{{q_{i} q_{j} }}{{r_{{ij}} }}} + \sum\limits_{{i \ne j}} {D_{{0,ij}} \left[ {\left( {\frac{{R_{{0,ij}} }}{{rij}}} \right)^{{12}} - 2\left( {\frac{{R_{{0,ij}} }}{{rij}}} \right)^{6} } \right]} .$$

**Figure 10:**
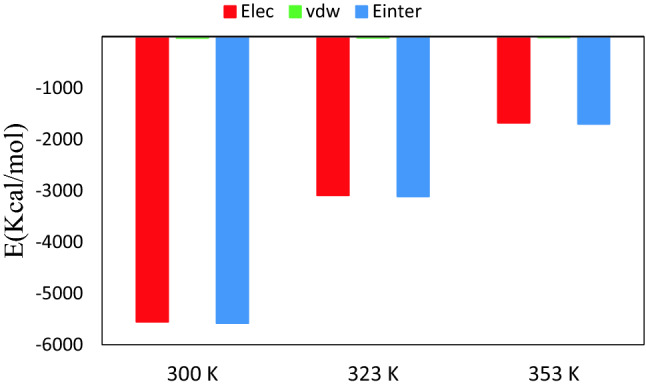
Intermolecular interaction energy, E_inter_, Van der Waals, E_vdW_, Electrostatic, E_coul_, between MEN: MEN for the binary mixtures of menthol and fatty acid with %LUA = 44 at different temperatures.

The values of the non-bonded energies are reported in Supplementary Table S3. It could be seen from Supplementary Table S3, Ecoul and E vdW are − 3235.06 kJ/mol and − 8.10 kJ/mol, respectively, in the binary mixtures CAP and MEN with %FAs = 44 at 323. These results point out that the electrostatic interaction energy is predominant between HBA and HBD. The vdW energy values are so trivial that they can be disregarded. The absolute value of the non-bonded energies shows that the interaction between LUA and MEN was significantly greater in the binary mixture with %FAs = 25 at 323 K than the other ratios. In term of energy, this percentage combination may be more suitable for the preparation of the eutectic mixture based on acid and menthol. However, interactions between two molecules reveal that temperature can be a more effective factor to be considered. Investigation of the interaction energy of MEN: MEN in the binary mixture with %FAs = 44 showed that the amount of energy was slightly decreased under various temperatures from 300 to 353 K (see Fig. 10). According to Supplementary Fig. S1, increasing the length of the alkyl chain of FAs may prevent further interaction between the HBA and HBD. The reduction of non-bonded energy is most likely related to the reduction of the partial charge on oxygen atoms FAs.

### Dynamical and transport properties

**Figure 11:**
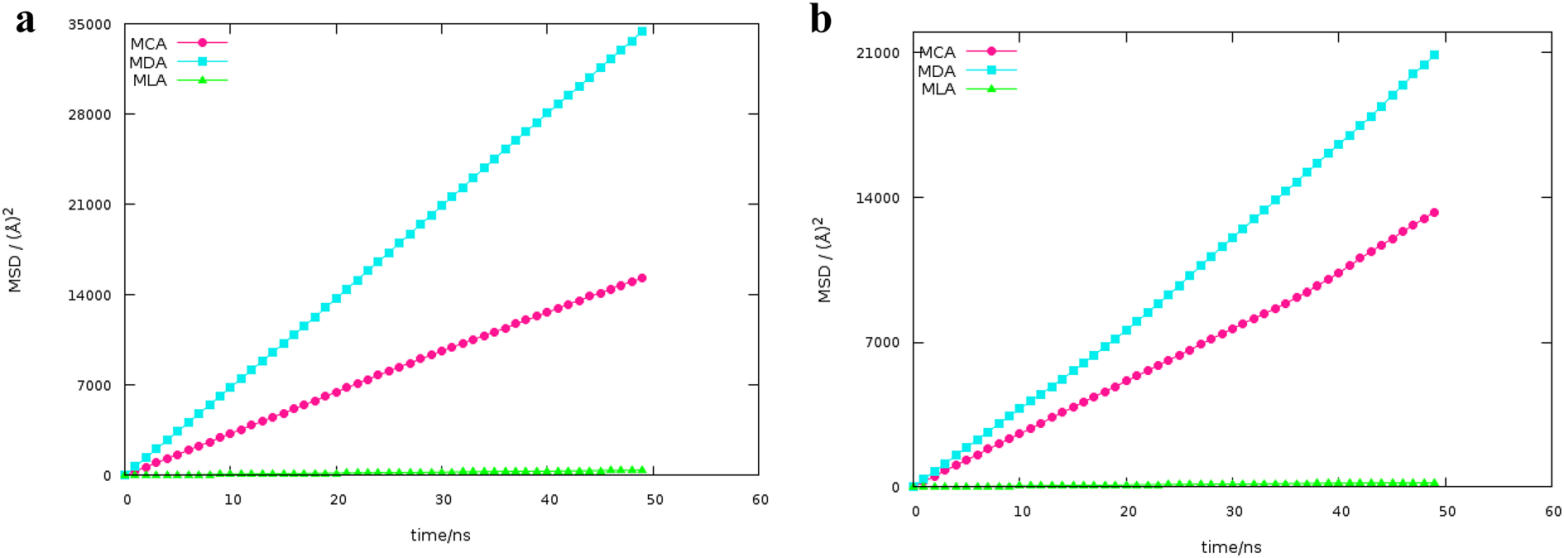
(**a**) The MSDs of the MEN for the binary mixtures of menthol and fatty acid with %FAs = 70 at 323 K, (**b**) The MSDs of the FAs for the binary mixtures of menthol and fatty acid with %FAs = 70 at 323 K.

The mean-squared displacements (MSDs) of species were measured to describe the migration of species^28^. The MSDs of species as a function of time were plotted for binary mixtures of FAs and MEN in the same ratio, see Fig. 11a,b. The low slope of the MSD curve of menthol molecules indicates a strong interaction between the two components in the binary mixture of menthol and Caprylic acid compared to the mixture of MDA. In comparison to MSD of DES molecules, due to interactions between FAs molecules which FAs molecules move in binary mixtures, the slope of the MSD curve of LUA molecules shows smaller value. The self-diffusivity coefficient is the macroscopic dynamical property which is calculated from MD simulation^29^. The self-diffusion coefficients of species were obtained from the slopes of the lines fitted to the MSD curves using the Einstein relation:

6 $$D=\underset{t\to \infty }{\mathit{lim}}\frac{\langle {\left|\Delta r\left(t\right)\right|}^{2}\rangle }{6t},$$

7 $$\left\langle {\Delta \left| {r\left( t \right)} \right|^{2} } \right\rangle = \frac{1}{N}\left\langle {\sum\limits_{{i = 1}}^{N} {\left| {r_{i}^{{COM}} \left( t \right) - r_{i}^{{COM}} \left( 0 \right)} \right|^{2} } } \right\rangle ,$$

where $\langle \Delta {\left|r\left(t\right)\right|}^{2}\rangle$ is the mean-square displacement (MSD) of species i. To ensure the measured values of self-diffusion coefficients, the diffusive regime was identified and sampling was performed in this regime^30^. The $\beta$ parameter was used to determine the location of the diffusive regime in the binary mixtures^31^. The beta parameter was used to the self-diffusivities of the species in DESs by Colina and coworkers^18^ and it is calculated according to:

8 $$\upbeta =\frac{d{\mathrm{log}}_{10}<\mathrm{\Delta r}({t)}^{2}>}{d{\mathrm{log}}_{10}t}.$$

The self-diffusion coefficient of the species was derived from the slope of the log–log plots of MSD in the range of 40 to 50 ns^18^. The calculated self-diffusion coefficient values from the diffusive regime are shown in Table 2. The self-diffusion coefficient obtained for the CAP was 0.92 Å^2^ ns^−1^ and 10.29 Å^2^ ns^−1^, respectively, in the binary mixture with %FAs = 44 at 300 K and 353 K. In general, the reduced viscosity at higher temperatures has led to more rapid migration of species in the diffusive regime. The self-diffusion coefficients of species in the binary mixtures with a molar percentage of 70% are ranked as MDA > MCA > MLA. The self-diffusion coefficient values for the CAP molecules were 4.29 Å^2^ ns^−1^ and 3.25 Å^2^ ns^−1^, respectively, in the binary mixture of FAs and MEN with %FAs = 20 K and 44 K, at 323 K. The self-diffusions of species decrease by adding the acid molecules into the simulation box. It should be noted that the remarkable changes were found in the binary mixtures of MCA, MDA, and MLA with %FAs = 44, 35, and 25. Reducing self-diffusion can be justified by intermolecular interactions.

**Table 2:** Calculated self-diffusion coefficients of species at different temperatures.

D Å^2^ ns^−1^			
T/K	MCA	CAP	MEN
	n OCT:n MEN		
323	200:800	4.29	5.40
300	440:560	0.92	0.89
323	440:560	3.25	3.90
353	440:560	10.27	12.13
323	700:300	5.34	6.09

T/K	MDA	DEC	MEN
	n DEC:n MEN		
323	200:800	6.35	11.68
323	350:650	0.83	1.40
323	700:300	9.15	19.76

T/K	MLA	LUA	MEN
	n LUA:n LUA		
323	200:800	0.83	1.39
300	250:750	0.095	0.16
323	250:750	0.60	1.21
353	250:750	3.63	7.41
323	700:300	0.070	1.13

### Vector reorientation dynamics (VRD)

**Figure 12:**
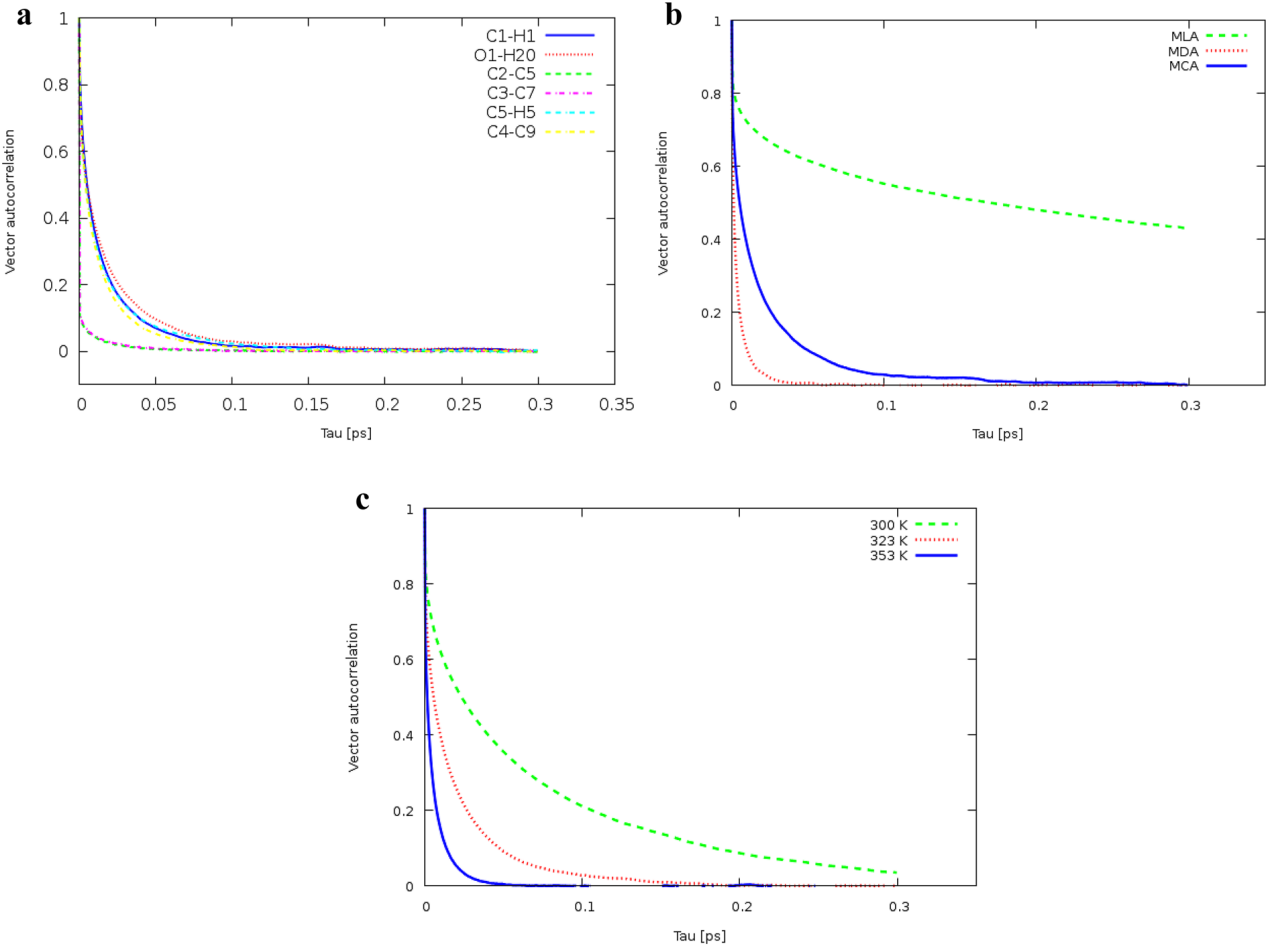
(**a**) Vector reorientation dynamics for bond vectors of MEN in the binary mixture of MCA with %CAP = 44 at 323 K, (**b**) Vector reorientation dynamics for bond vectors (HA–OA) of MEN in the binary mixture of MCA with %FAs = 70 at 323 K, (**c**) Vector reorientation dynamics for bond vectors (HA–OA) of MEN in the binary mixture of MLA with % LUA = 25 at different temperatures.

The vector reorientation dynamics is a common quantity that can be determined by both experimental techniques^32^ and molecular dynamics simulation methods. VRD is computed from Eq. (9),

9 $$VRD\left( \tau \right) = N.\left\langle {\sum\limits_{{t = 0}}^{{T - \tau }} {\vec{a}_{i} \left( t \right).\vec{a}_{i} \left( {t + \tau } \right)} } \right\rangle _{i} ,$$

where ${\overrightarrow{a}}_{i}\left(t\right)$ and ${\overrightarrow{a}}_{i}\left(t+\tau \right)$ are a vector selected on a molecule in step t and at the later time t + τ, respectively^33^. The reorientation dynamics of the bonds vector of the MEN molecules is displayed in Fig. 12a. As one can see, the reorientation of the bond vectors OA-HA in MEN is slower than the other bond vectors due to intermolecular interactions. As a result, the reorientation dynamics of HA–OA vectors is discussed in this section. For the reorientation of the HA–OA bond vectors, the blue and red solid lines and the green dashed lines indicate VRD in the binary mixtures of MCA, MDA, and MLA, respectively (see Fig. 12b). VRD of the HA–OA bond in Capric acid is considerably faster than this bond in the other acids. VRD of the HA–OA bond of Capric acid in the binary mixture of MCA with %FAs = 44 was the lowest one and increased at 353 K (see Fig. 12c). Interactions between FAs and MEN resulted in the lowest orientation velocity in the mixture of MCA with %CAP = 44. Similarly, the HA–OA bond has the fastest reorientation dynamic in the binary mixture with %LUA = 25 at 353 K compared to other temperatures. The slower orientation of the HA–OA bond can be related to the interaction of the hydrogen bond between the acid molecules that are reported in the “Hydrogen bond analysis”.

### Thermo-physical properties analysis

The shear viscosity η can be obtained from the xy elements of the molecular pressure tensor using the Green–Kubo expression^34^. Shear viscosity is given by

10 $$\eta = \frac{V}{{k_{B} T}}\int\limits_{0}^{\infty } {dt < P_{{xy}} \left( 0 \right)P_{{xy}} \left( t \right),}$$

where V and T are standing for the molar volume and the temperature, respectively, and K_B_ is the Boltzmann constant^35^.

11 $$P_{{xy}} = \sum\limits_{{i = 1}}^{N} {\frac{{p_{i}^{x} p_{i}^{y} }}{{m_{i} }}} - \sum\limits_{{i > j}}^{N} {\left( {x_{i} - x_{j} } \right)\frac{{\partial u_{{ij}} }}{{\partial y_{j} }}} ,$$

Here, ${p}_{i}^{x}$ and ${u}_{ij}$ indicate the momentum component and the interaction potential, respectively, and ${x}_{i}$ is a component of the radius vector^36^. The measured viscosity values are collected in Table 3. At 323 K, the values of η of the MCA and MDA binary mixtures are 121.6 and 104.51 MPa, respectively. Based on the results of the occupancy of the hydrogen bonding, the hydrogen bond network in the binary mixture of CAP and MEN is more stable than in the binary mixture of MDA. Thus, it can be concluded that the interactions between FAs and MEN molecules have an important effect on the shear viscosity of the binary mixtures. The effect of increasing FAs molecules on the shear viscosity of the binary mixtures was investigated by measuring the η at the different molar percent of FAs and the results reveal that adding of the FAs molecules into the binary mixtures increases the viscosity of all the mixtures of FAs and MEN due to bigger value of η in the binary mixtures containing 44% CAP is higher (52.451 MPa) than the binary mixture with the molar percentage of 20% (24.63 MPa). The values of the shear viscosity are in reasonable agreement with the hydrogen bonds result; therefore, the shear viscosity of the binary mixtures was decreased gradually during increasing of the temperature. Finally, It should be noted that the shear viscosities obtained for the binary mixtures of LUA and MEN with %FAs = 25 using MD simulation are close to the experimental value, with a difference of less than 2%^8^.

**Table 3:** The density distribution function (Dens) of the HA atom of MEN around the OA atom of FAs in the mixtures and the shear viscosity (η) of the binary mixtures from simulations.

T/K	MCA	Dens/distance (Å)		η/MPa
	n CAP:n MEN			
		R	Dens	
323	200:800	188.5	0.24	24.63
300	440:560	188.5	0.26	92.13
323	440:560	188.5	0.25	52.451
353	440:560	188.5	0.2	15.81
323	700:300	188.5	0.082	121.6

T/K	MDA			
	n DEC:n MEN			
323	200:800	192.5	0.11	40.14
323	350:650	192.5	0.19	96.49
323	700:300	192.5	0.026	104.51

T/K	MLA			
	n LUA:n MEN			
323	200:800	197.33	0.12	216.06
300	250:750	197.33	0.14	187.98
323	250:750	197.33	0.15	80.83
353	250:750	197.33	0.11	21.480
323	700:300	197.33	0.1	412.96

## Conclusion

In the present work, MD simulations was exploited to investigate the correlation between the structural and dynamical properties of hydrophobic eutectic mixtures. There is a strong interaction between FAs and MEN molecules in the binary mixtures of MCA, MDA, and MLA with %FAs = 44, 35, and 25, respectively. The maximum distribution between the two species of MEN and FAs in these ratios is estimated from structural analyzes such as RDF, CDF, SDF, and the density distribution function. The distance range of 2 to 3 Å and the range of angles between 130° and 180° of CDFs shows the maximum number of hydrogens bonds between the two species. A single fatty acid molecule can participate as a bridge in the binary mixture because it can accept H-bonds using the –COOH group. The increased temperatures lead to an increase in the migration of molecules, which we surmise that it is attributed to the changes in the intermolecular interactions. Furthermore, it was observed that the interaction between two species was reduced with the increase in the length of alkyl chain.

## Supplementary Information

Supplementary Information.
