# Supplementary material for: Structural and dynamic properties of eutectic mixtures based on menthol and fatty acids derived from coconut oil: a MD simulation study
Source: Sci Rep. 2022 Mar 25;12:5153. doi: 10.1038/s41598-022-09185-x (PMC8956646; doi:10.1038/s41598-022-09185-x)
Supplement: Supplementary file 1 — Supplementary Information. [file 41598_2022_9185_MOESM1_ESM.docx]

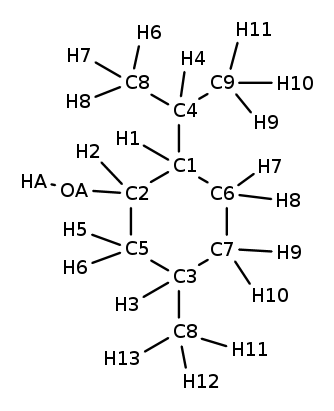


**Fig. S1**. Schematic of menthol (MEN) molecule with the main atomic labels.

Fig. S2. Intermolecular interaction energy, E_inter_ between MEN: FAs for the binary mixtures of menthol and fatty acid with %FAs = 70 at 323 K.

**Table S1**. Coordination Numbers of molecules around each other in the binary mixtures of FAs and MEN.

|  |  | **MCA** |  |
| --- | --- | --- | --- |
| **T/K** | **n MEN: n CAP** | **FAs.HA--FAs.OA** | **MEN.HA--MEN.OA** |
|  |  | **R N coord** | **R N coord** |
| 323 | 800 : 200 | 1.98 0.14 | 1.98 0.163 |
| 300 | 560 : 440 | 1.98 0.23 | 1.98 0.12 |
| 323 | 560 : 440 | 1.98 0.13 | 1.98 0.094 |
| 353 | 560 : 440 | 1.98 0.19 | 1.98 0.064 |
| 323 | 300 : 700 | 1.98 0.30 | 1.98 0.11 |
|  |  | **MDA** |  |
| **T/K** | **n MEN: n DEC** | **FAs.HA--FAs.OA** | **MEN.HA--MEN.OA** |
|  |  | **R N coord** | **R N coord** |
| 323 | 800 : 200 | 1.98 0.16 | 1.98 0.172 |
| 323 | 650 : 350 | 1.98 0.14 | 1.98 0.021 |
| 323 | 300 : 700 | 1.98 0.32 | 1.98 0.12 |
|  |  | **MLA** |  |
| **T/K** | **n MEN: n LUA** | **FAs.HA--FAs.OA** | **MEN.HA--MEN.OA** |
|  |  | **R N coord** | **R N coord** |
| 323 | 800 : 200 | 1.98 0.29 | 1.98 0.18 |
| 300 | 750 : 250 | 1.98 0.35 | 1.98 0.22 |
| 323 | 750 : 250 | 1.98 0.097 | 1.98 0.12 |
| 353 | 750 : 250 | 1.98 0.071 | 1.98 0.094 |
| 323 | 300 : 700 | 1.98 0.38 | 1.98 0.14 |

**Table S2**. The average number of hydrogen bonds between the species (N _avg_) in the binary mixtures.

|  |  | **MCA** | | |  |
| --- | --- | --- | --- | --- | --- |
| **T/K** | **n OCT: n MEN** | **MEN.HA – CAP.O** | | **CAP. HA-MEN.OA** | **MEN.HA- MEN.OA** |
| 323 | 200:800 | 97.7516 +/- 0.17 | | 102.472 +/- 0.25 | 133.812 +/- 0.19 |
| 300 | 440:560 | 206.197 +/- 0.20 | | 150.186 +/- 0.33 | 154.392 +/- 0.24 |
| 323 | 440:560 | 201.205 +/- 0.37 | | 139.522 +/- 0.24 | 111.643 +/- 0.19 |
| 353 | 440:560 | 179.798 +/- 0.29 | | 131.498 +/- 0.22 | 108.071 +/- 0.16 |
| 323 | 700:300 | 198.868 +/- 0.33 | | 137.589 +/- 0.25 | 122.3814 +/- 0.11 |
|  |  | **MDA** | | |  |
| **T/K** | **n OCT: n MEN** | **MEN.HA – DEC.O** | | **DEC. HA-MEN.OA** | **MEN.HA- MEN.OA** |
| 323 | 200:800 | 98.1353 +/- 0.27 | | 112.933 +/- 0.26 | 170.017 +/- 0.17 |
| 323 | 350:650 | 335.607 +/- 0.11 | | 127.766 +/- 0.16 | 141.777 +/-0.39 |
| 323 | 700:300 | 90.9477 +/- 0.24 | | 114.787 +/- 0.25 | 160.079 +/- 0.09 |
|  |  | | **MLA** | |  |
| **T/K** | **n OCT: n MEN** | | **MEN.HA – LUA.O** | **LUA. HA-MEN.OA** | **MEN.HA- MEN.OA** |
| 323 | 200:800 | 151.274 +/- 0.21 | | 144.373 +/- 0.12 | 256.272 +/- 0.28 |
| 300 | 250: 750 | 198.149 +/- 0.25 | | 160.912 +/- 0.09 | 378.937 +/- 0.18 |
| 323 | 250: 750 | 156.97 +/- 0.37 | | 158.39 +/- 0.12 | 218.401 +/- 0.32 |
| 353 | 250: 750 | 138.165 +/- 0.26 | | 102.61 +/- 0.19 | 182.91 +/- 0.30 |
| 323 | 700:300 | 118.569 +/- 0.19 | | 131.807 +/- 0.13 | 24.8.32 +/- 0.11 |

**Table S3**. Van der Waals, E_vdW_, Electrostatic, E_coul_, and intermolecular, E_inter_, Energies between HA _MEN_ -- O _FAs_ for simulated the binary mixtures.

|  |  | **MCA** |  |  |
| --- | --- | --- | --- | --- |
| T/K | **n MEN: n CAP** | **E_Vdw_ Kcal.mol ^-1^** | **E_coul_ Kcal.mol ^-1^** | **E_total_ Kcal.mol ^-1^** |
| 323 | 200:800 | -8.6444 +/- 0.03 | -3259.48 +/- 6.44 | -3268.12 +/- 6.86 |
| 300 | 440:560 | -8.6444 +/- 0.03 | -3459.48 +/- 6.44 | -3468.12 +/- 6.86 |
| 323 | 440:560 | -8.10258 +/- 0.02 | -3235.06 +/- 16.92 | -3243.16 +/- 17.87 |
| 353 | 440:560 | -5.98 +/- 0.03 | -2009.14 +/- 17.05 | -2015.12 +/- 16.21 |
|  |  | **MDA** |  |  |
|  | **n MEN: n DEC** |  |  |  |
|  |  |  |  |  |
| 323 | 200:800 | -5.96267 +/- 0.037 | -2303.25 +/- 24.93 | -2309.21 +/- 24.1 |
|  |  | **MLA** |  |  |
|  | **n MEN: n LUA** |  |  |  |
|  |  |  |  |  |
| 323 | 200:800 | -5.03504 +/- 0.02 | -1728.85 +/- 19.88 | -1733.28 +/- 20.05 |

**Table S4**. MD simulation density results, ρ, for the mixtures of menthol and fatty acids.

|  | **MCA** |  |
| --- | --- | --- |
| **T/K** | **n MEN: n CAP** | **ρ / g·cm^-3^** |
| 323 | 800:200 | 0.92057 |
| 300 | 440:560 | 1.194 |
| 323 | 440:560 | 0.94701 |
| 353 | 440:560 | 0.1233 |
| 323 | 300:700 | 0.9274 |
|  | **MDA** |  |
|  | **n MEN: n DEC** |  |
| 323 | 800:200 | 0.9085 |
| 323 | 650:350 | 0.9180 |
| 323 | 300:700 | 0.8622 |
|  | **MLA** |  |
|  | **n MEN: n LUA** |  |
| 323 | 800:200 | 0.9745 |
| 300 | 750:250 | 1.4244 |
| 323 | 750:250 | 0.9910 |
| 353 | 750:250 | 0.8502 |
| 323 | 300:700 | 0.9135 |

| **Table S5**. Force field parameters for fatty acids molecules . |  |
| --- | --- |
| \| **Force field parameter (Caprylic acid)** \| \|  \| \| \| **Value** \| \| \|  \| \| \| --- \| --- \| --- \| --- \| --- \| --- \| --- \| --- \| --- \| --- \| \| **Atom** \| \| **Atom type** \| \| \| **charge** \| \| \|  \| \| \| C1 \| \| CG2O2 \| \| \| 0.7769 \| \| \|  \| \| \| O1 \| \| OG311 \| \| \| -0.6104 \| \| \|  \| \| \| O2 \| \| OG2D1 \| \| \| -0.6953 \| \| \|  \| \| \| HO1 \| \| HGP1 \| \| \| 0.4704 \| \| \|  \| \| \| H83, H82 ,H81 \| \| HGA3 \| \| \| 0.0153 \| \| \|  \| \| \| C2 \| \| CG321 \| \| \| 0.0315 \| \| \|  \| \| \| C3 \| \| CG321 \| \| \| 0.0021 \| \| \|  \| \| \| H21, H22 \| \| HGA2 \| \| \| -0.0011 \| \| \|  \| \| \| C4 \| \| CG321 \| \| \| -0.0008 \| \| \|  \| \| \| C5 \| \| CG321 \| \| \| 0.0098 \| \| \|  \| \| \| C8 \| \| CG321 \| \| \| -0.074 \| \| \|  \| \| \| C6 \| \| CG321 \| \| \| -0.007 \| \| \|  \| \| \| C7 \| \| CG321 \| \| \| -0.0473 \| \| \|  \| \| \| H31, H32 \| \| HGA2 \| \| \| -0.0011 \| \| \|  \| \| \| H41, H42 \| \| HGA2 \| \| \| 0.0028 \| \| \|  \| \| \| H51, H52 \| \| HGA2 \| \| \| 0.0019 \| \| \|  \| \| \| H61, H62 \| \| HGA2 \| \| \| -0.0058 \| \| \|  \| \| \| H71, H72 \| \| HGA2 \| \| \| 0.0399 \| \| \|  \| \| \| **Bond stretching parameters** \| \| **Kb[kcal/mole/A**2]** \| \| \| **b 0 [**Å **]** \| \| \|  \| \| \| CG2O2 OG311 \| \| 390.94 \| \| \| 1.394 \| \| \|  \| \| \| CG2O2 OG2D1 \| \| 825.876 \| \| \| 1.232 \| \| \|  \| \| \| CG2O2 CG321 \| \| 304.771 \| \| \| 1.524 \| \| \|  \| \| \| OG311 HGP1 \| \| 474.378 \| \| \| 0.987 \| \| \|  \| \| \| CG321 HGA2 \| \| 340.691 \| \| \| 1.098 \| \| \|  \| \| \| CG321 CG321 \| \| 291.999 \| \| \| 1.529 \| \| \|  \| \| \| CG321 CG331 \| \| 290 \| \| \| 1.5 \| \| \|  \| \| \| CG331 HGA3 \| \| 330 \| \| \| 1.08 \| \| \|  \| \| \| **Angle bending parameters** \| \|  \| \| \|  \| \| \|  \| \| \| **Angle type** \| \| 𝐊𝛉[𝐤𝐜𝐚𝐥/𝐦𝐨𝐥𝐞/𝐫𝐚𝐝 ∗∗ 𝟐 ] \| \| \| 𝜽**[°]** \| \| \|  \| \| \| CG2O2 CG321 CG321 \| \| 54.82 \| \| \| 111.734 \| \| \|  \| \| \| CG2O2 CG321 CG321 \| \| 54.82 \| \| \| 111.734 \| \| \|  \| \| \| CG2O2 OG311 HGP1 \| \| 107.738 \| \| \| 110.566 \| \| \|  \| \| \| OG311 CG2O2 CG321 \| \| 68.064 \| \| \| 113.798 \| \| \|  \| \| \| OG311 CG2O2 OG2D1 \| \| 112.842 \| \| \| 126.671 \| \| \|  \| \| \| OG2D1 CG2O2 CG321 \| \| 76.635 \| \| \| 128.735 \| \| \|  \| \| \| OG2D1 CG2O2 CG321 \| \| 76.635 \| \| \| 128.735 \| \| \|  \| \| \| CG321 CG321 HGA2 \| \| 46.582 \| \| \| 109.271 \| \| \|  \| \| \| HGA2 CG321 HGA2 \| \| 36.029 \| \| \| 107.109 \| \| \|  \| \| \| **Torsional parameters** \| \|  \| \| \|  \| \| \|  \| \| \| **Dihedral type** \| \| **K chi [kcal/mole]** \| \| \| **n [multiplicity]** \| \| \| **delta [°]** \| \| \| CG321 CG321 CG321 CG321 \| \| 1.52 \| \| \| 3 \| \| \| 0 \| \| \| CG321 CG321 CG321 HGA2 \| \| 0.254 \| \| \| 2 \| \| \| 180 \| \| \| CG321 CG2O2 OG311 HGP1 \| \| 1.95 \| \| \| 2 \| \| \| 180 \| \| \| OG2O1 CG2O2 CG321 CG321 \| \| 0.908 \| \| \| 2 \| \| \| 180 \| \| \| CG2O2 CG321 CG321 HGA2 \| \| 0.7 \| \| \| 3 \| \| \| 0 \| \| \| CG321 CG321 CG331 HGA3 \| \| 0.25 \| \| \| 2 \| \| \| 180 \| \| \| HGA2 CG321 CG321 HGA2 \| \| 0.287 \| \| \| 2 \| \| \| 180 \| \| \| OG311 CG2O2 CG321 HGA2 \| \| 0.762 \| \| \| 2 \| \| \| 180 \| \| \| CG321 CG321 CG321 CG331 \| \| 1.022 \| \| \| 3 \| \| \| 0 \| \| \| HGA2 CG321 CG331 HGA3 \| \| 2.507 \| \| \| 2 \| \| \| 180 \| \| \| OG2D1 CG2O2 CG321 HGA2 \| \| 1.939 \| \| \| 1 \| \| \| 180 \| \| \| OG2D1 CG2O2 OG311 HGP1 \| \| 1.782 \| \| \| 2 \| \| \| 180 \| \| \| HGA2 CG321 CG321 CG331 \| \| 0.2 \| \| \| 2 \| \| \| 180 \| \| \| **Force field parameter (Decanoic acid)** \| \|  \| \| \| **Value** \| \| \| \|  \| \| **Atom** \| \| **Atom type** \| \| \| **charge** \| \| \| \|  \| \| O1 \| \| OG311 \| \| \| 0.6103 \| \| \| \|  \| \| O2 \| \| OG2D1 \| \| \| -0.6948 \| \| \| \|  \| \| C10 \| \| CG331 \| \| \| -0.0817 \| \| \| \|  \| \| H18, H19, H20 \| \| HGA3 \| \| \| 0.0166 \| \| \| \|  \| \| C9 \| \| CG321 \| \| \| -0.0624 \| \| \| \|  \| \| H16,H17 \| \| HGA2 \| \| \| -0.0034 \| \| \| \|  \| \| C8 \| \| CG321 \| \| \| 0.0219 \| \| \| \|  \| \| H14,H15 \| \| HGA2 \| \| \| 0.0006 \| \| \| \|  \| \| H12, H13 \| \| HGA2 \| \| \| -0.004 \| \| \| \|  \| \| H10, H11 \| \| HGA2 \| \| \| -0.0083 \| \| \| \|  \| \| H8, H9 \| \| HGA2 \| \| \| -0.0065 \| \| \| \|  \| \| H6, H7 \| \| HGA2 \| \| \| 0.0006 \| \| \| \|  \| \| H4, H5 \| \| HGA2 \| \| \| -0.0034 \| \| \| \|  \| \| H2, H3 \| \| HGA2 \| \| \| 0.0043 \| \| \| \|  \| \| H1 \| \| HGP1 \| \| \| 0.4536 \| \| \| \|  \| \| C7 \| \| CG321 \| \| \| -0.0003 \| \| \| \|  \| \| C6 \| \| CG321 \| \| \| 0.0669 \| \| \| \|  \| \| C5 \| \| CG321 \| \| \| 0.0148 \| \| \| \|  \| \| C4 \| \| CG321 \| \| \| 0.0425 \| \| \| \|  \| \| C3 \| \| CG321 \| \| \| 0.0061 \| \| \| \|  \| \| C2 \| \| CG321 \| \| \| 0.0387 \| \| \| \|  \| \| C1 \| \| CG2D2 \| \| \| 0.696 \| \| \| \|  \| \| **Bond stretching parameters** \| \|  \| \| \|  \| \| \| \|  \| \| **Bond type** \| \| **Kb[kcal/mole/A**2]** \| \| \| **b 0 [**Å **]** \| \| \| \|  \| \| CG2D2 OG311 \| \| 402.768 \| \| \| 1.389 \| \| \| \|  \| \| CG2D2 OG2D1 \| \| 850.802 \| \| \| 1.228 \| \| \| \|  \| \| CG2D2 CG321 \| \| 301.998 \| \| \| 1.517 \| \| \| \|  \| \| OG311 HGP1 \| \| 479.172 \| \| \| 0.986 \| \| \| \|  \| \| CG321 HGA2 \| \| 341.026 \| \| \| 1.098 \| \| \| \|  \| \| CG321 CG321 \| \| 290.227 \| \| \| 1.526 \| \| \| \|  \| \| CG321 CG331 \| \| 293.186 \| \| \| 1.526 \| \| \| \|  \| \| CG331 HGA3 \| \| 356.453 \| \| \| 1.094 \| \| \| \|  \| \| **Angle bending parameters** \| \|  \| \| \|  \| \| \| \|  \| \| **Angle type** \| \| 𝐊𝛉[𝐤𝐜𝐚𝐥/𝐦𝐨𝐥𝐞/𝐫𝐚𝐝 ∗∗ 𝟐 ] \| \| \| 𝜽**[°]** \| \| \| \|  \| \| CG2D2 CG321 CG321 \| \| 76.052 \| \| \| 111.095 \| \| \| \|  \| \| CG2D2 CG321 HGA2 \| \| 57.956 \| \| \| 108.115 \| \| \| \|  \| \| CG2D2 OG311 HGP1 \| \| 148.329 \| \| \| 107.649 \| \| \| \|  \| \| OG311 CG2D2 CG321 \| \| 67.245 \| \| \| 112.749 \| \| \| \|  \| \| OG311 CG2D2 OG2D1 \| \| 134.434 \| \| \| 125.527 \| \| \| \|  \| \| OG2D1 CG2D2 CG321 \| \| 85.493 \| \| \| 127.885 \| \| \| \|  \| \| CG321 CG321 CG321 \| \| 52.561 \| \| \| 112.831 \| \| \| \|  \| \| CG321 CG321 HGA2 \| \| 53.264 \| \| \| 109.484 \| \| \| \|  \| \| HGA2 CG321 HGA2 \| \| 24.709 \| \| \| 107.074 \| \| \| \|  \| \| CG321 CG321 CG331 \| \| 79.927 \| \| \| 112.693 \| \| \| \|  \| \| CG321 CG331 HGA3 \| \| 59.42 \| \| \| 111.202 \| \| \| \|  \| \| HGA2 CG321 CG331 \| \| 45.913 \| \| \| 110.272 \| \| \| \|  \| \| HGA3 CG331 HGA3 \| \| 50.525 \| \| \| 107.973 \| \| \| \|  \| \| **Torsional parameters** \| \|  \| \| \|  \| \| \| \|  \| \| **Dihedral type** \| \| **K chi [kcal/mole]** \| \| \| **n [multiplicity]** \| \| \| \| **delta [°]** \| \| \| CG321 CG321 CG321 CG321 \| \| 2.997 \| \| \| 2 \| \| \| \| 180 \| \| CG321 CG321 CG321 HGA2 \| \| 2.944 \| \| \| 2 \| \| \| \| 180 \| \| CG321 CG2O2 OG311 HGP1 \| \| 2.979 \| \| \| 3 \| \| \| \| 180 \| \| OG2O1 CG2D2 CG321 CG321 \| \| 2.93 \| \| \| 3 \| \| \| \| 0 \| \| CG2O2 CG321 CG321 HGA2 \| \| 2.958 \| \| \| 2 \| \| \| \| 180 \| \| CG321 CG321 CG331 HGA3 \| \| 2.652 \| \| \| 3 \| \| \| \| 180 \| \| HGA2 CG321 CG321 HGA2 \| \| 1.022 \| \| \| 3 \| \| \| \| 0 \| \| OG311 CG2O2 CG321 HGA2 \| \| 2.992 \| \| \| 3 \| \| \| \| 180 \| \| CG321 CG321 CG321 CG331 \| \| 2.868 \| \| \| 3 \| \| \| \| 0 \| \| HGA2 CG321 CG331 HGA3 \| \| 2.507 \| \| \| 2 \| \| \| \| 180 \| \| OG2D1 CG2D2 CG321 HGA2 \| \| 0.362 \| \| \| 2 \| \| \| \| 0 \| \| OG2D1 CG2D2 OG311 HGP1 \| \| 2.706 \| \| \| 3 \| \| \| \| 180 \| \| HGA2 CG321 CG321 CG331 \| \| 2.966 \| \| \| 2 \| \| \| \| 180 \| \| **Force field parameter (Lauric acid)** \| \|  \| **Value** \| \| \| \|  \| \| \| **Atom** \| \| **Atom type** \| **charge** \| \| \| \|  \| \| \| O1 \| \| OG311 \| -0.589282 \| \| \| \|  \| \| \| O2 \| \| OG2D1 \| -0.642 \| \| \| \|  \| \| \| H1 \| \| HGP1 \| 0.4702 \| \| \| \|  \| \| \| C1 \| \| CG2O2 \| 0.7782 \| \| \| \|  \| \| \| C2 \| \| CG321 \| -0.0453 \| \| \| \|  \| \| \| C3 \| \| CG321 \| -0.0106 \| \| \| \|  \| \| \| C4 \| \| CG321 \| 0.0085 \| \| \| \|  \| \| \| C5 \| \| CG321 \| 0.0091 \| \| \| \|  \| \| \| C6 \| \| CG321 \| 0.0636 \| \| \| \|  \| \| \| C7 \| \| CG321 \| 0.0149 \| \| \| \|  \| \| \| C8 \| \| CG321 \| 0.0235 \| \| \| \|  \| \| \| C9 \| \| CG321 \| 0.023818 \| \| \| \|  \| \| \| C10 \| \| CG321 \| 0.0103 \| \| \| \|  \| \| \| C11 \| \| CG321 \| 0.0331 \| \| \| \|  \| \| \| C12 \| \| CG331 \| -0.0766 \| \| \| \|  \| \| \| H2,H3 \| \| HGA2 \| 0.0392 \| \| \| \|  \| \| \| H4,H5 \| \| HGA2 \| 0.0111 \| \| \| \|  \| \| \| H6,H7 \| \| HGA2 \| -0.0048 \| \| \| \|  \| \| \| H8,H9 \| \| HGA2 \| 0.0005 \| \| \| \|  \| \| \| H10,H11 \| \| HGA2 \| -0.005 \| \| \| \|  \| \| \| H12, H13 \| \| HGA2 \| -0.0066 \| \| \| \|  \| \| \| H14, H15 \| \| HGA2 \| -0.0103 \| \| \| \|  \| \| \| H16,H17 \| \| HGA2 \| -0.0059 \| \| \| \|  \| \| \| H18,H19 \| \| HGA2 \| -0.0004 \| \| \| \|  \| \| \| H20,H21 \| \| HGA2 \| -0.0023 \| \| \| \|  \| \| \| H22, H23, H24 \| \| HGA3 \| 0.0154 \| \| \| \|  \| \| \| **Bond stretching parameters** \| \|  \|  \| \| \| \|  \| \| \| **Bond type** \| **Kb[kcal/mole/A**2]** \| \| \| **b 0 [**Å **]** \| \| \|  \| \| \| CG2O2 OG311 \| \| 403.862 \| 1.391 \| \| \| \|  \| \| \| CG2D2 OG2D1 \| \| 847.272 \| 1.227 \| \| \| \|  \| \| \| CG2O2 CG321 \| \| 306.05 \| 1.521 \| \| \| \|  \| \| \| OG311 HGP1 \| \| 493.07 \| 0.984 \| \| \| \|  \| \| \| CG321 HGA2 \| \| 342.293 \| 1.097 \| \| \| \|  \| \| \| CG321 CG321 \| \| 293.064 \| 1.525 \| \| \| \|  \| \| \| CG321 CG331 \| \| 310.226 \| 1.524 \| \| \| \|  \| \| \| CG331 HGA3 \| \| 355.129 \| 1.095 \| \| \| \|  \| \| \| **Angle bending parameters** \| \|  \|  \| \| \| \|  \| \| \| **Angle type** \| \| 𝐊𝛉[𝐤𝐜𝐚𝐥/𝐦𝐨𝐥𝐞/𝐫𝐚𝐝 ∗∗ 𝟐 ] \| \| \| 𝜽**[°]** \| \|  \| \| \| CG2O2 CG321 CG321 \| \| 75.431 \| 111.054 \| \| \| \|  \| \| \| CG2O2 CG321 HGA2 \| \| 61.258 \| 108.398 \| \| \| \|  \| \| \| CG2O2 OG311 HGP1 \| \| 111.631 \| 109.571 \| \| \| \|  \| \| \| OG311 CG2O2 CG321 \| \| 97.105 \| 113.246 \| \| \| \|  \| \| \| OG311 CG2O2 OG2D1 \| \| 137.077 \| 125.449 \| \| \| \|  \| \| \| OG2D1 CG2O2 CG321 \| \| 64.117 \| 128.448 \| \| \| \|  \| \| \| CG321 CG321 CG321 \| \| 61.79 \| 112.685 \| \| \| \|  \| \| \| CG321 CG321 HGA2 \| \| 46.801 \| 109.298 \| \| \| \|  \| \| \| HGA2 CG321 HGA2 \| \| 33.349 \| 106.595 \| \| \| \|  \| \| \| CG321 CG321 CG331 \| \| 42.662 \| 112.599 \| \| \| \|  \| \| \| CG321 CG331 HGA3 \| \| 44.876 \| 111.165 \| \| \| \|  \| \| \| HGA2 CG321 CG331 \| \| 65.169 \| 109.993 \| \| \| \|  \| \| \| HGA3 CG331 HGA3 \| \| 43.117 \| 108.026 \| \| \| \|  \| \| \| **Torsional parameters** \| \|  \|  \| \| \| \|  \| \| \| **Dihedral type** \| \| **K chi [kcal/mole]** \| **n [multiplicity]** \| \| \| \| **delta [°]** \| \| \| CG321 CG321 CG321 CG321 \| \| 2.991 \| 2 \| \| \| \| 0 \| \| \| CG321 CG321 CG321 HGA2 \| \| 2.894 \| 2 \| \| \| \| 180 \| \| \| CG321 CG2O2 OG311 HGP1 \| \| 2.65 \| 3 \| \| \| \| 0 \| \| \| OG2D1 CG2O2 CG321 CG321 \| \| 1.866 \| 3 \| \| \| \| 180 \| \| \| CG2O2 CG321 CG321 CG321 \| \| 2.921 \| 3 \| \| \| \| 0 \| \| \| CG321 CG321 CG331 HGA3 \| \| 2.712 \| 3 \| \| \| \| 0 \| \| \| HGA2 CG321 CG321 HGA2 \| \| 2.796 \| 2 \| \| \| \| 0 \| \| \| OG311 CG2O2 CG321 HGA2 \| \| 2.509 \| 3 \| \| \| \| 0 \| \| \| CG321 CG321 CG321 CG331 \| \| 2.524 \| 3 \| \| \| \| 0 \| \| \| HGA2 CG321 CG331 HGA3 \| \| 2.19 \| 3 \| \| \| \| 180 \| \| \| OG2D1 CG2O2 CG321 HGA2 \| \| 2.214 \| 2 \| \| \| \| 0 \| \| \| OG2D1 CG2O2 OG311 HGP1 \| \| 2.375 \| 3 \| \| \| \| 0 \| \| \| HGA2 CG321 CG321 CG331 \| \| 2.967 \| 3 \| \| \| \| 0 \| \| \| H11 \| \| HGA3 \| \| \| \| 0.0557 \| \| \|  \| \| \| \| C8 \| \| CG331 \| \| \| \| -0.2161 \| \| \|  \| \| \| \| H6, H7, H8 \| \| HGA3 \| \| \| \| 0.0557 \| \| \|  \| \| \| \| **Bond stretching parameters** \| \|  \| \| \| \|  \| \| \|  \| \| \| \| **Bond type** \| \| **Kb[kcal/mole/A**2]** \| \| \| \| **b 0 [Å ]** \| \| \|  \| \| \| \| CG2R61 OG311 \| \| 456.963 \| \| \| \| 1.376 \| \| \|  \| \| \| \| CG2R61 CG2R61 \| \| 354.793 \| \| \| \| 1.395 \| \| \|  \| \| \| \| OG311 HGP1 \| \| 560.901 \| \| \| \| 0.971 \| \| \|  \| \| \| \| CG2R61 CG312 \| \| 387.523 \| \| \| \| 1.523 \| \| \|  \| \| \| \| CG312 HGA1 \| \| 368.295 \| \| \| \| 1.094 \| \| \|  \| \| \| \| CG312 CG331 \| \| 299.126 \| \| \| \| 1.534 \| \| \|  \| \| \| \| CG2R61 HGR61 \| \| 377.705 \| \| \| \| 1.086 \| \| \|  \| \| \| \| CG2R61 CG331 \| \| 327.881 \| \| \| \| 1.513 \| \| \|  \| \| \| \| CG331 HGA3 \| \| 361.239 \| \| \| \| 1.097 \| \| \|  \| \| \| \| CG331 HGA1 \| \| 365.233 \| \| \| \| 1.091 \| \| \|  \| \| \| \| **Angle bending parameters** \| \|  \| \| \| \|  \| \| \|  \| \| \| \| **Angle type** \| \| 𝐊𝛉[𝐤𝐜𝐚𝐥/𝐦𝐨𝐥𝐞/𝐫𝐚𝐝 ∗∗ 𝟐 ] \| \| \| \| 𝜽[°] \| \| \|  \| \| \| \| CG2R61 CG2R61 HGR61 \| \| 72.235 \| \| \| \| 119.392 \| \| \|  \| \| \| \| CG2R61 CG2R61 CG2R61 \| \| 296.752 \| \| \| \| 119.841 \| \| \|  \| \| \| \| CG2R61 CG2R61 CG312 \| \| 284.05 \| \| \| \| 121.665 \| \| \|  \| \| \| \| CG2R61 OG311 HGP1 \| \| 144.203 \| \| \| \| 108.475 \| \| \|  \| \| \| \| OG311 CG2R61 CG2R61 \| \| 296.272 \| \| \| \| 119.281 \| \| \|  \| \| \| \| CG2R61 CG312 CG331 \| \| 218.79 \| \| \| \| 112.551 \| \| \|  \| \| \| \| CG2R61 CG312 HGA1 \| \| 73.166 \| \| \| \| 105.602 \| \| \|  \| \| \| \| CG312 CG331 HGA3 \| \| 135.597 \| \| \| \| 111.094 \| \| \|  \| \| \| \| CG312 CG331 HGA1 \| \| 55.199 \| \| \| \| 111.597 \| \| \|  \| \| \| \| CG2R61 CG2R61 CG331 \| \| 112.319 \| \| \| \| 120.931 \| \| \|  \| \| \| \| CG2R61 CG331 HGA3 \| \| 56.262 \| \| \| \| 110.983 \| \| \|  \| \| \| \| HGA1 CG312 CG331 \| \| 70.186 \| \| \| \| 106.049 \| \| \|  \| \| \| \| HGA3 CG331 HGA1 \| \| 50.155 \| \| \| \| 107.774 \| \| \|  \| \| \| \| CG331 CG312 CG331 \| \| 95.349 \| \| \| \| 112.612 \| \| \|  \| \| \| \| HGA1 CG331 HGA1 \| \| 59.079 \| \| \| \| 107.374 \| \| \|  \| \| \| \| HGA3 CG331 HGA3 \| \| 87.864 \| \| \| \| 107.489 \| \| \|  \| \| \| \| **Torsional parameters** \| \|  \| \| \| \|  \| \| \|  \| \| \| \| **Dihedral type** \| \| **K chi [kcal/mole]** \| \| \| \| **n [multiplicity]** \| \| \| **delta [°]** \| \| \| \| OG311 CG2R61 CG2R61 CG2R61 \| \| 1.44 \| \| \| \| 2 \| \| \| 0 \| \| \| \| HGR61 CG2R61 CG2R61 HGR61 \| \| 1.581 \| \| \| \| 3 \| \| \| 0 \| \| \| \| CG312 CG2R61 CG2R61 HGR61 \| \| 2.001 \| \| \| \| 3 \| \| \| 180 \| \| \| \| HGR61 CG2R61 CG2R61 CG331 \| \| 1.092 \| \| \| \| 3 \| \| \| 180 \| \| \| \| HGA1 CG312 CG331 HGA1 \| \| 0.426 \| \| \| \| 3 \| \| \| 0 \| \| \| \| CG2R61 CG2R61 CG312 CG331 \| \| 1.377 \| \| \| \| 3 \| \| \| 0 \| \| \| \| CG2R61 CG2R61 CG2R61 CG2R61 \| \| 2.424 \| \| \| \| 3 \| \| \| 180 \| \| \| \| HGA1 CG312 CG331 HGA3 \| \| 1.086 \| \| \| \| 2 \| \| \| 0 \| \| \| \| CG2R61 CG2R61 OG311 HGP1 \| \| 2.326 \| \| \| \| 3 \| \| \| 180 \| \| \| \| CG2R61 CG2R61 CG331 HGA3 \| \| 1.928 \| \| \| \| 2 \| \| \| 180 \| \| \| \| CG2R61 CG312 CG331 HGA1 \| \| 1.081 \| \| \| \| 3 \| \| \| 180 \| \| \| \| CG331 CG2R61 CG2R61 CG2R61 \| \| 19.95 \| \| \| \| 3 \| \| \| 0 \| \| \| \| CG2R61 CG312 CG331 HGA3 \| \| 1.66 \| \| \| \| 2 \| \| \| 180 \| \| \| \| CG2R61 CG2R61 CG312 HGA1 \| \| 2.18 \| \| \| \| 3 \| \| \| 180 \| \| \| \| CG331 CG312 CG331 HGA1 \| \| 1.812 \| \| \| \| 2 \| \| \| 180 \| \| \| \| OG311 CG2R61 CG2R61 HGR61 \| \| 0.833 \| \| \| \| 3 \| \| \| 0 \| \| \| \| OG311 CG2R61 CG2R61 CG312 \| \| 1.813 \| \| \| \| 2 \| \| \| 180 \| \| \| \| CG331 CG312 CG331 HGA3 \| \| 0.009 \| \| \| \| 3 \| \| \| 0 \| \| \| \| CG2R61 CG2R61 CG2R61 CG312 \| \| 2.846 \| \| \| \| 3 \| \| \| 0 \| \| \| \| CG2R61 CG2R61 CG2R61 HGR61 \| \| 0.954 \| \| \| \| 2 \| \| \| 180 \| \| \| | |
